# Social security cuts and life expectancy: a longitudinal analysis of local authorities in England, Scotland and Wales

**DOI:** 10.1136/jech-2023-220328

**Published:** 2023-11-07

**Authors:** Rosie Seaman, David Walsh, Christina Beatty, Gerry McCartney, Ruth Dundas

**Affiliations:** 1 MRC/CSO Social and Public Health Sciences Unit, University of Glasgow, Glasgow, UK; 2 Glasgow Centre for Population Health, Glasgow, UK; 3 Centre for Regional Economic and Social Research, Sheffield Hallam University, Sheffield, UK; 4 School of Health and Wellbeing, University of Glasgow, Glasgow, UK

**Keywords:** death, health inequalities, mortality

## Abstract

**Background:**

The UK Government’s ‘welfare reform’ programme included reductions to social security payments, phased in over the financial years 2011/2012–2015/2016. Previous studies of social security cuts and health outcomes have been restricted to analysing single UK countries or single payment types (eg, housing benefit). We examined the association between all social security cuts fully implemented by 2016 and life expectancy, for local authorities in England, Scotland and Wales.

**Methods:**

Our unit of analysis was 201 upper tier local authorities (unitary authorities and county councils: 147 in England, 32 in Scotland, 22 in Wales). Our exposure was estimated social security loss per head of the working age population per year for each local authority, calculated against the baseline in 2010/2011. The primary outcome was annual life expectancy at birth between the calendar years 2012 and 2016 (year lagged following exposure). We used a panel regression approach with fixed effects.

**Results:**

Social security cuts implemented by 2016 were estimated to be £475 per head of the working age population in England, £390 in Scotland and £490 in Wales since 2010/2011. During the study period, there was either no improvement or only marginal increases in national life expectancy. Social security loss and life expectancy were significantly associated: an estimated £100 decrease in social security per head of working age population was associated with a 1-month reduction in life expectancy.

**Conclusions:**

Social security cuts, at the UK local authority level, were associated with lower life expectancy. Further research should examine causality.

**WHAT IS ALREADY KNOWN ON THIS TOPIC**

- Following the introduction of austerity in the UK in 2010, previous long-term improvements in life expectancy stagnated or reversed, with increasing death rates observed among more deprived populations and older ages.

**WHAT THIS STUDY ADDS**

- We examined the association between all social security cuts implemented by March 2016 and life expectancy for all local authorities in England, Scotland, and Wales.
- We found that every £100 decrease in social security spending per head of the working age population per year compared to 2011/2012, was associated with an estimated 1-month loss in life expectancy.

**HOW THIS STUDY MIGHT AFFECT RESEARCH, PRACTICE OR POLICY**

- Social security cuts may be negatively associated with declining population health outcomes in the UK, future research should examine whether this relationship is causal.

## Introduction

Life expectancy is a marker of population health and for more than a century, many countries have seen overall increases. In Europe, the major exceptions to increases have been temporary and due to pandemics (Spanish Influenza and COVID-19), wars or particular social crises (the break-up of the USSR).^1 2^ Yet after 2010, national life expectancy in the UK stalled and there were decreases in life expectancy among more socioeconomically deprived populations and older age groups.^3–11^

A large body of research,^12–16^ including a recently published critical assessment of evidence,^17^ highlights the potential role UK Government ‘austerity’ may have played. Austerity policies were introduced after the 2008 recession and aimed to reduce overall government spending by c.£85 billion.^18^

Austerity may have impacted population health in two important ways. First, by removing or reducing public services through cuts to local government funding. Second, by cutting individual incomes when tens of billions of pounds were cut from the UK social security budget and social security payments were reduced.^17 19 20^

Social security is defined as ‘the protection that a society provides to individuals and households to ensure access to healthcare and to guarantee income security, particularly in cases of old age, unemployment, sickness, invalidity, work injury, maternity or loss of a breadwinner’.^21^ Most healthcare is free to access in the UK, and the key element of social security is a minimum income security safety net.

Income is a determinant of health and social security cuts were concentrated on individuals and households claiming benefits and tax credits, who already experience an increased mortality risk.^22^ The potential causal pathways from social security loss to adverse health and mortality include increased poverty, stress related biological responses to poverty (with stress being a risk factor for chronic disease), adverse impacts on mental health, and health damaging coping mechanisms in terms of alcohol and drug use.^17^

A number of analyses have demonstrated the association between public spending cuts and health outcomes.^13 14 23–25^ Analyses of social security cuts have been limited either in terms of analysing single types of social security payments,^11 15^ geographical coverage (eg, only England^11^ or Scotland^12^), or have focused on non-mortality outcomes.^26 27^ We aimed to examine the association between all major social security cuts that were fully implemented and life expectancy in England, Scotland and Wales.

We used local authorities as our unit of analysis to account for the uneven geographical distribution of the scale of social security cuts, which tended to disproportionately impact the more socioeconomically deprived areas.^20^ Local authorities in the UK are the local government and administrative bodies in each defined geographical area. We were able to examine whether the association between social security cuts and life expectancy differed by UK nation.^28^

## Methods

### Unit of analysis

Our unit of analysis was 201 upper tier local authorities (unitary authorities and county councils: 147 in England, 32 in Scotland, 22 in Wales). We excluded five local authorities in England from the statistical analyses. Three were dropped due to boundary differences between the mortality and population data geographies (Bournemouth, Christchurch and Poole) and the social security loss data geographies (Dorset and Poole). Two were dropped because of small populations (City of London and Isles of Scilly).

### Exposure variable

The exposure variable was the decrease in social security per head of working age population for local authorities. We had annual exposure data for the financial years 2011/2012 to 2015/2016, with the amount lost per year increasing as social security cuts were phased in.

During the study period, there were different social security payment types, each subject to different eligibility and entitlement criteria. As part of the UK Government’s programme of ‘welfare reform’, changes were made to different social security payment types including Housing Benefit, non-dependant deductions, Benefit Cap, Council Tax Support, Personal Independence Payment, Employment and Support Allowance, Child Benefit, Tax Credits, CPI and 1% up-rating (limiting the annual increase in value of benefits), and Universal Credit (work allowances and waiting times).

We did not have access to data on individual level changes in social security payments. Instead, we estimated the average annual social security payment loss per head of the working age population residing in each local authority. These are an updated set of data that have been used in a range of other analyses.^15 29^ We estimated the local authority level effect of social security reforms, in millions of pounds, using a range of published government statistics. The statistics used for our estimates included treasury estimates of the anticipated saving arising from each element of the reforms, published in the budget or in the government’s autumn statement, impact assessments that government departments publish for most elements of the reforms, claimant numbers and expenditure, by local authority, published by the Department for Work and Pensions (DWP) and HM Revenue and Customs (HMRC).^30^

To improve interpretability, we scaled the exposure variable in the regression analyses to represent every estimated £100 lost per head of the working age population. The social security data used in the analyses are provided in online supplemental material S1 and were updated for this study by Sheffield Hallam University.^20 30^

10.1136/jech-2023-220328.supp1 Supplementary data

**Figure 1:**
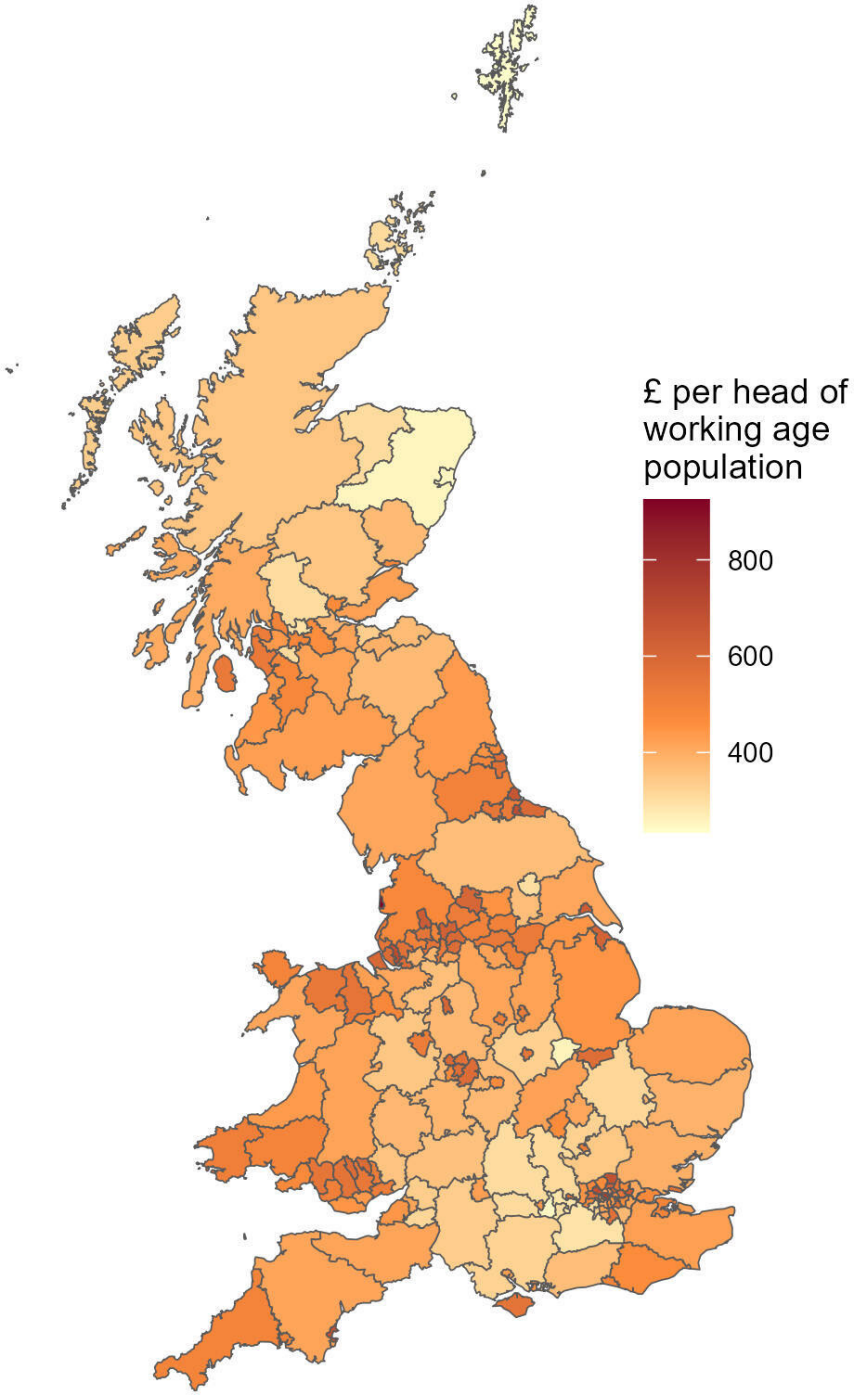
Local authority map of social security loss (£ per head of the working age population) fully implemented by March 2016.

Figure 1 is a map showing estimated loss in social security per head of the working age population for each local authority, fully implemented by March 2016.

### Outcome variables

The primary outcome was period life expectancy at birth, by sex, for each local authority. Life expectancy was calculated from death counts and mid-year population estimates. All data are publicly available on request from national agencies (for England and Wales, the Office for National Statistics via https://www.nomisweb.co.uk/ and for Scotland the National Records of Scotland).

We estimated mortality rates from deaths counts and population estimates, as 3-year rolling averages. We constructed abridged life tables using standard age groups (<1 year, 1–5 years, 5–9 years up to 85+ years) and estimated life expectancy at birth using standard life table notation.^31^ In our analyses, we used annual life expectancy estimates for the calendar years 2012–2016 to capture mortality in the year following exposure. The middle calendar year is the reference year for all the data used (eg, 2012 includes data for 2011–2013). Our annual life expectancy estimates were relatively stable over the study period, with only a few of the smallest local authorities demonstrating random annual fluctuations. We used the Human Mortality Database (HMD) national estimates as an external comparator for our own national estimates. Our national life expectancy estimates were comparable but not identical to the HMD. This is to be expected as different data were used, with HMD estimates being available for England and Wales combined. We plotted the years 2007 and 2018, as these were all the years of comparable data we had for local authorities. Note that we only used life expectancy estimates for the years 2012 to 2016 as the outcome variables in our statistical analyses.

**Figure 2:**
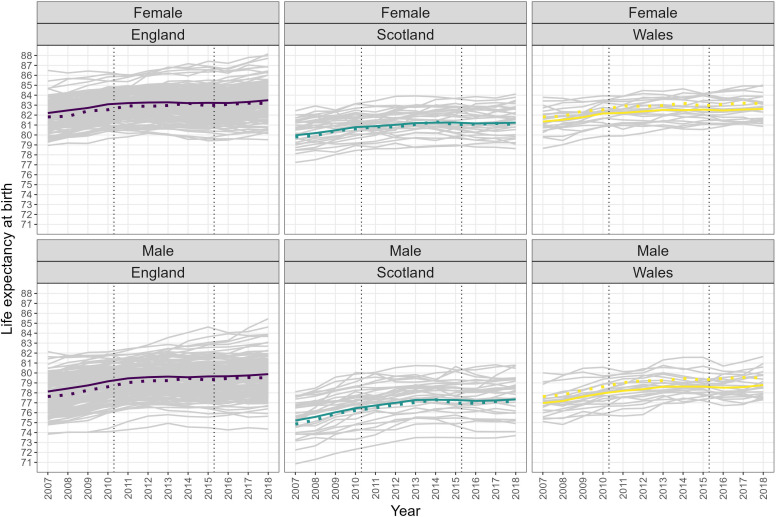
Trends in life expectancy at birth for each local authority (grey lines) and national estimates (coloured lines) with Human Mortality Database national estimates for comparison (coloured dots).

Figure 2 shows life expectancy trends for each local authority in England, Scotland, and Wales (grey lines) and national estimates (coloured lines) compared with national estimates from the HMD (coloured dots). Male and female life expectancy in England was consistently higher than in Wales and, particularly, Scotland.

### Statistical analyses

We used a fixed-effects panel regression to examine the association between exposure and outcome, fitting regressions separately for males and females.

For each of the local authorities, we used social security data for each financial year (starting in 2011/2012 and ending in 2015/2016), and absolute life expectancy at birth data for each year starting in 2012 and ending in 2016. We ended our analysis in 2016 as most of the UK Government’s programme of ‘welfare reform’ had been fully implemented by this point. The main model aimed to estimate the association between the weighted average social security payment loss (£ per head of the working aged population (16–64 years old)) and the weighted life expectancy for all local authorities in each country (capturing the year following exposure).

We included fixed effects for each local authority to account for unobserved confounding variables that would vary between local authorities, but that would be constant over the time period. This means the models we estimated used within-local-authority variation over time to estimate the relationship between social security loss and life expectancy, not the differences between local authorities.

We weighted the models by the sex-specific population size of each local authority within each year. We included an interaction term between country and cumulative social security payment loss to see if any association differed for Scotland and Wales compared with England. Autocorrelation between residuals was observed: to correct for this, all models reported here included robust SEs.^32^

### Sensitivity analyses

We carried out three sensitivity checks (online supplemental materials S2–S5). We examined a longer (2-year) time lag between exposure (social security loss) and outcome (life expectancy). We hypothesised that the association between social security loss and life expectancy would be stronger for working ages than older ages and ran our main regression model using life expectancy at age 65 years as an alternative outcome variable. As a further sensitivity analysis, we also used lifespan variation (edagger) as an alternative outcome.^33^

10.1136/jech-2023-220328.supp2 Supplementary data

All data management and analyses were completed using R V.4.3.0 (R code are available via the Open Science Framework DOI 10.17605/OSF.IO/WJMK2).

## Results

### Descriptive analyses

Table 1 gives the weighted averages across all local authorities in each country for the exposure variable (annual social security loss per head of the working age population compared with baseline (£)) and the outcome variable (life expectancy at birth).

**Table 1:** Social security loss (£ per head of the working age population per year) and average life expectancy (years) for each country (weighted by all local authorities)

Country	Year	Social security loss (£) (exposure)	Male life expectancy (outcome)	Female life expectancy (outcome)
England	2011	75.11	–	–
England	2012	182.95	79.29	83.15
England	2013	310.91	79.37	83.19
England	2014	397.43	79.32	83.12
England	2015	475.09	79.41	83.15
England	2016	–	79.43	83.15
Scotland	2011	62.32	–	–
Scotland	2012	150.50	77.42	81.29
Scotland	2013	244.36	77.65	81.46
Scotland	2014	320.25	77.65	81.51
Scotland	2015	387.47	77.65	81.51
Scotland	2016	–	77.58	81.44
Wales	2011	79.12	–	–
Wales	2012	189.46	78.42	82.39
Wales	2013	312.09	78.72	82.54
Wales	2014	406.42	78.70	82.55
Wales	2015	490.75	78.68	82.62
Wales	2016		78.57	82.54

The average estimated social security cuts per head of the working age population, which were fully implemented by 2016, and in that year amounted to £475 in England, £390 in Scotland and £490 in Wales.

Average estimated national life expectancy, when weighted across all local authorities, increased only marginally for each country. For example, life expectancy increased by only 0.14 years between 2012 and 2016 for males in England; in Scotland, there was an increase of 0.16 years, and in Wales it was 0.15 years. For females in England, life expectancy did not change between 2012 and 2016; in Scotland there was an increase of 0.15 years and in Wales it was 0.14 years.

### Fixed-effects panel regression model

Table 2 presents the results of the social security loss fixed effects regression model. For both males and females, a negative association between social security loss and life expectancy was observed, with the magnitude similar for both sexes. For females, on average, every £100 loss per head of the working age population was associated with a 0.09-year decrease in life expectancy (95% CI −0.13 to −0.04). The equivalent main effect for males was 0.08 years for every £100 loss. In crude terms, these figures equate to an estimated 1-month decrease in life expectancy associated with every estimated £100 loss in social security per head of the working age population. With an average loss of £475 in England per head of the working age population across all local authorities, this would be associated with a life expectancy being nearly 5 months lower given the scale of social security cuts between 2011/2012 and 2015/2016.

**Table 2:** Fixed effects regression results—the effect of cumulative social security cuts on life expectancy at birth

Sex	Term	Ex estimates	95% CI lower	95% CI upper	P value
Female	(Intercept)	83.53	83.47	83.59	0.00
Female	Year (mortality)	0.07	0.03	0.12	0.00
Female	Social security loss	0.09	0.13	0.04	0.00
Female	Scotland	2.48	2.67	2.29	0.00
Female	Wales	1.79	2.10	1.48	0.00
Female	Social security loss×Scotland	0.03	0.01	0.07	0.16
Female	Social security loss×Wales	0.04	0.01	0.08	0.01
Male	(Intercept)	80.05	79.73	80.36	0.00
Male	Year (mortality)	0.10	0.05	0.16	0.00
Male	Social security loss	0.08	0.14	0.02	0.01
Male	Scotland	3.19	3.60	2.77	0.00
Male	Wales	1.54	1.89	1.19	0.00
Male	Social security loss×Scotland	0.01	0.03	0.06	0.63
Male	Social security loss×Wales	0.02	0.03	0.07	0.51

The main effect of country in our regression model indicated that life expectancy was lower in Scotland (females: −2.48 years, males: −3.88 years) and Wales (females: −1.79 years, males: −1.54 years) compared with England. The interaction term for social security loss and country indicated that the negative effect of social security loss on life expectancy was largest in England, compared with Scotland and Wales but this was insignificant.

The results of the robustness checks are in online supplemental materials S2–S5. Extending the time lag to 2 years reduced the effect size of the estimated social security cuts for males and was no longer statistically significant. The effect size for social security remained similar and statistically significant for females. It notably altered the results for the interaction term between country and social security loss (online supplemental material S2). No statistically significant associations between social security loss and life expectancy at 65 years were observed (online supplemental material S3). We hypothesised that social security cuts would have had a stronger association with working age mortality to which life expectancy at birth is more sensitive than remaining life expectancy at age 65. We found no statistically significant association between social security cuts and lifespan variation (online supplemental materials S4 and S5), which may reflect the fact that lifespan variation was calculated within local authorities rather than reflecting inequalities between areas.^33^

## Discussion

### Summary of findings

In every decade prior to the early 2010s, life expectancy in the UK increased. In the 1980s, males gained 1.8 and females gained 1.4 years. In the 1990s males gained 2.2 and females gained 1.5 years. In the first decade of the 2000s, males gained 2.1 and females gained 2.3 years.^34^ This contrasts with the changes shown here for the years 2012–2016. Males only gained 0.5 years and females have only gained 0.4 years. The relatively small gains we have captured in this study are prior to any COVID-19 effects. Our study found that across all local authorities, social security payment cuts—one key component of the UK Government’s austerity policies—were negatively associated with life expectancy.

### Strengths and limitations

We have built on previous analyses by including data for all local authorities in England, Scotland and Wales. As such, our data are representative of total populations and do not represent samples. We demonstrate the importance of considering all social security cuts implemented.^35^ Households often receive a combination of social security payments rather than one payment in isolation.

We capture the importance of geography. The magnitude of social security cuts in any local authority is related to many factors—the demographics of local population, household size, household tenure, social security benefit type and local employment context. We used the most up to date, annual estimates for social security cuts which are more detailed than previously used.^30^

Finally, life expectancy is a robust health indicator that is not associated with the potential biases shown with some self-reported measures of health.^36^

Limitations of our study are that alternative population health outcomes (eg, cause-specific mortality, mental health and morbidity) might have been more sensitive to the exposure. Although fixed-effect analyses accounts for time-invariant confounding, it is possible that there is unmeasured time-varying confounding in the model. We did not have individual level data available on the amount of social security payments lost. Having individual level data would enable causality to be modelled. We only covered a short period of time and longer-term impacts should be monitored.

### Interpretations and implications

Reductions in social security are not the only possible explanation for the UK’s stalling life expectancy and geographical inequalities. Other proposed explanations have included the role of certain health conditions (eg, influenza, dementia, cardiovascular disease), previous increases in obesity prevalence, various demographic shifts and data and methodological issues. However, a critical assessment of all that evidence, published in 2022, suggested that while a small proportion of the changed mortality and life expectancy may well be attributable to earlier changes to obesity levels, the biggest cause of the stalling was indeed UK Government austerity measures introduced since 2010.^17^

Returning to the hypothesis that social security cuts may explain stalling life expectancy, Richardso *et al* used different data sets to model the association between UK social security and tax changes and life expectancy in Scotland. They estimated a loss of 20 and 23 weeks (approximately 0.4 years) in female and male life years respectively between 2010/11 and 2021/2213.^22^ These are similar to our findings of an estimated loss of approximately 1 month of life expectancy for every estimated £100 in social security per head of the working age population (with our estimated total loss for Scotland being around £390 per head of the working age population). Other studies have examined cuts to specific social security elements and/or have focused on more specific health outcomes than overall life expectancy. For example, Koltai *et al* demonstrated an association between disability-related social security reductions and increased rates of drugs-related deaths,^15^ while Loopstra *et al* showed that a 1% cut in pension credits (a benefit given to pensioners on low incomes) was associated with an increase in mortality among the elderly.^11^ There is also evidence of detrimental effects on mental health from different social security cuts such as income support restrictions^26^ and Universal Credit.^27^ All these studies demonstrate a negative association between reductions in social security and population health outcomes.

Additional studies examined the association between health and cuts to local government funding (the other main component of austerity policies in the UK) as opposed to cuts to social security. Alexiou *et al* showed that across all local authorities in England, each £100 cut in spending (per person per annum) was associated with a decrease of approximately 1.2 and 1.3 months of life expectancy for females and males between 2013 and 2016.^13^ Martin *et al* examined the association between department-specific local authority funding and mortality in England, demonstrating that a 1% increase in healthcare, social care and public health funding was associated with 0.5%, 0.3% and 0.0% decreases, respectively, in mortality. As a consequence, cuts to funding in England between 2010/2011 and 2014/2015 were associated with an extra 57 500 deaths.^14^ Stokes *et al* showed that a 1% per capita decrease in total service expenditure in England was associated with a 0.1% increase in multimorbidity (two or more long-term health conditions) in the population.^25^ All these studies used local authority funding, which represents a different potential mechanism between austerity and mortality. Beyond these UK studies, there is an increasing amount of international evidence of the negative association between austerity policies and health in high-income countries.^37–39^

## Conclusions

UK Government austerity policies are often found to be associated with decreases or stalls in life expectancy. Future research should use individual level data to evaluate the causal relationship between social security cuts and mortality. This is of particular importance, given the accelerating cost-of-living crisis in the UK and its potential implications for population health.^40^

## Data Availability

Data are available on reasonable request. All data are publicly available upon request from national agencies (for England and Wales, the Office for National Statistics via https://www.nomisweb.co.uk/, and for Scotland the National Records of Scotland). Social security data used for the exposure are provided as online supplementary material S1.
