# Supplementary material for: Social security cuts and life expectancy: a longitudinal analysis of local authorities in England, Scotland and Wales
Source: J Epidemiol Community Health. 2023 Nov 7;78(2):82–7. doi: 10.1136/jech-2023-220328 (PMC10850624; doi:10.1136/jech-2023-220328)
Supplement: Supplementary data [file jech-2023-220328supp001.pdf]

ons_code_	council_na	years_welf	years_welf	years_welf	cuml_lossp	tier_code_upper
S12000033	Aberdeen (	2011_2012	2011	2012	38.89843	S12
S12000033	Aberdeen (	2012_2013	2012	2013	94.99356	S12
S12000033	Aberdeen (	2013_2014	2013	2014	160.2946	S12
S12000033	Aberdeen (	2014_2015	2014	2015	209.3292	S12
S12000033	Aberdeen (	2015_2016	2015	2016	251.9588	S12
S12000034	Aberdeensl	2011_2012	2011	2012	39.17534	S12
S12000034	Aberdeensl	2012_2013	2012	2013	98.87555	S12
S12000034	Aberdeensl	2013_2014	2013	2014	169.0585	S12
S12000034	Aberdeensl	2014_2015	2014	2015	214.4214	S12
S12000034	Aberdeensl	2015_2016	2015	2016	253.5752	S12
S12000041	Angus	2011_2012	2011	2012	61.52832	S12
S12000041	Angus	2012_2013	2012	2013	149.1515	S12
S12000041	Angus	2013_2014	2013	2014	238.8887	S12
S12000041	Angus	2014_2015	2014	2015	309.3605	S12
S12000041	Angus	2015_2016	2015	2016	371.9224	S12
S12000035	Argyll and I	2011_2012	2011	2012	66.96799	S12
S12000035	Argyll and I	2012_2013	2012	2013	162.9101	S12
S12000035	Argyll and I	2013_2014	2013	2014	257.7734	S12
S12000035	Argyll and I	2014_2015	2014	2015	334.8902	S12
S12000035	Argyll and I	2015_2016	2015	2016	403.9129	S12
E09000002	Barking anc	2011_2012	2011	2012	100.558	E09
E09000002	Barking anc	2012_2013	2012	2013	243.423	E09
E09000002	Barking anc	2013_2014	2013	2014	424.8639	E09
E09000002	Barking anc	2014_2015	2014	2015	542.8975	E09
E09000002	Barking anc	2015_2016	2015	2016	648.3168	E09
E09000003	Barnet	2011_2012	2011	2012	70.68915	E09
E09000003	Barnet	2012_2013	2012	2013	175.3044	E09
E09000003	Barnet	2013_2014	2013	2014	298.9793	E09
E09000003	Barnet	2014_2015	2014	2015	385.3704	E09
E09000003	Barnet	2015_2016	2015	2016	461.8092	E09
E08000016	Barnsley	2011_2012	2011	2012	81.65192	E08
E08000016	Barnsley	2012_2013	2012	2013	195.1968	E08
E08000016	Barnsley	2013_2014	2013	2014	345.6378	E08
E08000016	Barnsley	2014_2015	2014	2015	443.9361	E08
E08000016	Barnsley	2015_2016	2015	2016	533.7094	E08
E06000022	Bath and N	2011_2012	2011	2012	49.51944	E06
E06000022	Bath and N	2012_2013	2012	2013	121.9869	E06
E06000022	Bath and N	2013_2014	2013	2014	210.4278	E06
E06000022	Bath and N	2014_2015	2014	2015	265.03	E06
E06000022	Bath and N	2015_2016	2015	2016	314.4542	E06
E06000055	Bedford UA	2011_2012	2011	2012	66.23659	E06
E06000055	Bedford UA	2012_2013	2012	2013	161.9693	E06
E06000055	Bedford UA	2013_2014	2013	2014	268.4435	E06
E06000055	Bedford UA	2014_2015	2014	2015	343.9688	E06
E06000055	Bedford UA	2015_2016	2015	2016	410.782	E06
E09000004	Bexley	2011_2012	2011	2012	62.94261	E09

E09000004	Bexley	2012_2013	2012	2013	157.4371	E09
E09000004	Bexley	2013_2014	2013	2014	278.9911	E09
E09000004	Bexley	2014_2015	2014	2015	352.7625	E09
E09000004	Bexley	2015_2016	2015	2016	417.4805	E09
E08000025	Birminghar	2011_2012	2011	2012	91.99744	E08
E08000025	Birminghar	2012_2013	2012	2013	219.7203	E08
E08000025	Birminghar	2013_2014	2013	2014	373.6919	E08
E08000025	Birminghar	2014_2015	2014	2015	481.4128	E08
E08000025	Birminghar	2015_2016	2015	2016	579.1934	E08
E06000008	Blackburn \	2011_2012	2011	2012	103.188	E06
E06000008	Blackburn \	2012_2013	2012	2013	245.1272	E06
E06000008	Blackburn \	2013_2014	2013	2014	411.6976	E06
E06000008	Blackburn \	2014_2015	2014	2015	531.0016	E06
E06000008	Blackburn \	2015_2016	2015	2016	639.6722	E06
E06000009	Blackpool U	2011_2012	2011	2012	154.3661	E06
E06000009	Blackpool U	2012_2013	2012	2013	369.5128	E06
E06000009	Blackpool U	2013_2014	2013	2014	586.9408	E06
E06000009	Blackpool U	2014_2015	2014	2015	761.4153	E06
E06000009	Blackpool U	2015_2016	2015	2016	925.1201	E06
W0600001	Blaenau Gv	2011_2012	2011	2012	89.93872	W06
W0600001	Blaenau Gv	2012_2013	2012	2013	211.8267	W06
W0600001	Blaenau Gv	2013_2014	2013	2014	360.8754	W06
W0600001	Blaenau Gv	2014_2015	2014	2015	477.8536	W06
W0600001	Blaenau Gv	2015_2016	2015	2016	582.4427	W06
E08000001	Bolton	2011_2012	2011	2012	90.12256	E08
E08000001	Bolton	2012_2013	2012	2013	215.5491	E08
E08000001	Bolton	2013_2014	2013	2014	348.8453	E08
E08000001	Bolton	2014_2015	2014	2015	451.7729	E08
E08000001	Bolton	2015_2016	2015	2016	543.4723	E08
E06000036	Bracknell F	2011_2012	2011	2012	44.56049	E06
E06000036	Bracknell F	2012_2013	2012	2013	113.7302	E06
E06000036	Bracknell F	2013_2014	2013	2014	205.1282	E06
E06000036	Bracknell F	2014_2015	2014	2015	256.1604	E06
E06000036	Bracknell F	2015_2016	2015	2016	300.3872	E06
E08000032	Bradford	2011_2012	2011	2012	98.77539	E08
E08000032	Bradford	2012_2013	2012	2013	236.3879	E08
E08000032	Bradford	2013_2014	2013	2014	391.2256	E08
E08000032	Bradford	2014_2015	2014	2015	501.6872	E08
E08000032	Bradford	2015_2016	2015	2016	602.5812	E08
E09000005	Brent	2011_2012	2011	2012	113.3407	E09
E09000005	Brent	2012_2013	2012	2013	277.7655	E09
E09000005	Brent	2013_2014	2013	2014	448.3872	E09
E09000005	Brent	2014_2015	2014	2015	577.8933	E09
E09000005	Brent	2015_2016	2015	2016	691.5016	E09
W0600001	Bridgend /	2011_2012	2011	2012	81.51916	W06
W0600001	Bridgend /	2012_2013	2012	2013	194.7959	W06
W0600001	Bridgend /	2013_2014	2013	2014	318.0206	W06

W0600001	Bridgend /	2014_2015	2014	2015	417.5028	W06
W0600001	Bridgend /	2015_2016	2015	2016	506.7635	W06
E06000043	Brighton ar	2011_2012	2011	2012	81.66409	E06
E06000043	Brighton ar	2012_2013	2012	2013	200.7573	E06
E06000043	Brighton ar	2013_2014	2013	2014	319.0968	E06
E06000043	Brighton ar	2014_2015	2014	2015	408.3782	E06
E06000043	Brighton ar	2015_2016	2015	2016	490.3173	E06
E06000023	Bristol, City	2011_2012	2011	2012	66.87062	E06
E06000023	Bristol, City	2012_2013	2012	2013	161.8225	E06
E06000023	Bristol, City	2013_2014	2013	2014	264.7147	E06
E06000023	Bristol, City	2014_2015	2014	2015	344.4146	E06
E06000023	Bristol, City	2015_2016	2015	2016	415.8553	E06
E09000006	Bromley	2011_2012	2011	2012	54.41931	E09
E09000006	Bromley	2012_2013	2012	2013	138.601	E09
E09000006	Bromley	2013_2014	2013	2014	253.8969	E09
E09000006	Bromley	2014_2015	2014	2015	318.4547	E09
E09000006	Bromley	2015_2016	2015	2016	375.1348	E09
E10000002	Buckinghar	2011_2012	2011	2012	47.77106	E10
E10000002	Buckinghar	2012_2013	2012	2013	122.0863	E10
E10000002	Buckinghar	2013_2014	2013	2014	216.9818	E10
E10000002	Buckinghar	2014_2015	2014	2015	267.1372	E10
E10000002	Buckinghar	2015_2016	2015	2016	309.4807	E10
E08000002	Bury	2011_2012	2011	2012	79.53397	E08
E08000002	Bury	2012_2013	2012	2013	193.114	E08
E08000002	Bury	2013_2014	2013	2014	315.3897	E08
E08000002	Bury	2014_2015	2014	2015	407.0955	E08
E08000002	Bury	2015_2016	2015	2016	488.8775	E08
W0600001	Caerphilly /	2011_2012	2011	2012	83.89969	W06
W0600001	Caerphilly /	2012_2013	2012	2013	200.3696	W06
W0600001	Caerphilly /	2013_2014	2013	2014	338.423	W06
W0600001	Caerphilly /	2014_2015	2014	2015	442.4372	W06
W0600001	Caerphilly /	2015_2016	2015	2016	535.1151	W06
E08000033	Calderdale	2011_2012	2011	2012	83.57181	E08
E08000033	Calderdale	2012_2013	2012	2013	201.9191	E08
E08000033	Calderdale	2013_2014	2013	2014	338.2818	E08
E08000033	Calderdale	2014_2015	2014	2015	433.9366	E08
E08000033	Calderdale	2015_2016	2015	2016	520.8626	E08
E10000003	Cambridge:	2011_2012	2011	2012	48.57607	E10
E10000003	Cambridge:	2012_2013	2012	2013	122.1526	E10
E10000003	Cambridge:	2013_2014	2013	2014	213.9088	E10
E10000003	Cambridge:	2014_2015	2014	2015	267.7597	E10
E10000003	Cambridge:	2015_2016	2015	2016	314.0698	E10
E09000007	Camden	2011_2012	2011	2012	64.73425	E09
E09000007	Camden	2012_2013	2012	2013	161.3525	E09
E09000007	Camden	2013_2014	2013	2014	269.6202	E09
E09000007	Camden	2014_2015	2014	2015	342.3626	E09
E09000007	Camden	2015_2016	2015	2016	406.8331	E09

W0600001	Cardiff / Ca	2011_2012	2011	2012	70.07104	W06
W0600001	Cardiff / Ca	2012_2013	2012	2013	168.8793	W06
W0600001	Cardiff / Ca	2013_2014	2013	2014	278.4572	W06
W0600001	Cardiff / Ca	2014_2015	2014	2015	362.6288	W06
W0600001	Cardiff / Ca	2015_2016	2015	2016	437.3081	W06
W0600001	Carmarthe	2011_2012	2011	2012	80.03774	W06
W0600001	Carmarthe	2012_2013	2012	2013	191.0215	W06
W0600001	Carmarthe	2013_2014	2013	2014	313.1065	W06
W0600001	Carmarthe	2014_2015	2014	2015	407.7925	W06
W0600001	Carmarthe	2015_2016	2015	2016	492.4865	W06
E06000056	Central Bec	2011_2012	2011	2012	48.46882	E06
E06000056	Central Bec	2012_2013	2012	2013	121.5415	E06
E06000056	Central Bec	2013_2014	2013	2014	221.4742	E06
E06000056	Central Bec	2014_2015	2014	2015	276.0334	E06
E06000056	Central Bec	2015_2016	2015	2016	324.5129	E06
W0600000	Ceredigion	2011_2012	2011	2012	74.41225	W06
W0600000	Ceredigion	2012_2013	2012	2013	178.143	W06
W0600000	Ceredigion	2013_2014	2013	2014	279.439	W06
W0600000	Ceredigion	2014_2015	2014	2015	361.6728	W06
W0600000	Ceredigion	2015_2016	2015	2016	435.6762	W06
E06000049	Cheshire Ea	2011_2012	2011	2012	56.97158	E06
E06000049	Cheshire Ea	2012_2013	2012	2013	140.4624	E06
E06000049	Cheshire Ea	2013_2014	2013	2014	242.3283	E06
E06000049	Cheshire Ea	2014_2015	2014	2015	305.4708	E06
E06000049	Cheshire Ea	2015_2016	2015	2016	362.0304	E06
E06000050	Cheshire W	2011_2012	2011	2012	62.87729	E06
E06000050	Cheshire W	2012_2013	2012	2013	153.5181	E06
E06000050	Cheshire W	2013_2014	2013	2014	271.7827	E06
E06000050	Cheshire W	2014_2015	2014	2015	344.6576	E06
E06000050	Cheshire W	2015_2016	2015	2016	410.6534	E06
S12000005	Clackmann	2011_2012	2011	2012	72.46834	S12
S12000005	Clackmann	2012_2013	2012	2013	173.3084	S12
S12000005	Clackmann	2013_2014	2013	2014	285.728	S12
S12000005	Clackmann	2014_2015	2014	2015	381.2351	S12
S12000005	Clackmann	2015_2016	2015	2016	464.2357	S12
W0600000	Conwy / Cc	2011_2012	2011	2012	90.99086	W06
W0600000	Conwy / Cc	2012_2013	2012	2013	217.5616	W06
W0600000	Conwy / Cc	2013_2014	2013	2014	340.4285	W06
W0600000	Conwy / Cc	2014_2015	2014	2015	442.9515	W06
W0600000	Conwy / Cc	2015_2016	2015	2016	535.4251	W06
E06000052	Cornwall U	2011_2012	2011	2012	80.1252	E06
E06000052	Cornwall U	2012_2013	2012	2013	192.1334	E06
E06000052	Cornwall U	2013_2014	2013	2014	319.4021	E06
E06000052	Cornwall U	2014_2015	2014	2015	409.7224	E06
E06000052	Cornwall U	2015_2016	2015	2016	492.9727	E06
E06000047	County Dur	2011_2012	2011	2012	80.2258	E06
E06000047	County Dur	2012_2013	2012	2013	192.6149	E06

E06000047	County Dur	2013_2014	2013	2014	318.2709	E06
E06000047	County Dur	2014_2015	2014	2015	413.704	E06
E06000047	County Dur	2015_2016	2015	2016	499.2954	E06
E08000026	Coventry	2011_2012	2011	2012	77.8263	E08
E08000026	Coventry	2012_2013	2012	2013	188.2214	E08
E08000026	Coventry	2013_2014	2013	2014	300.2644	E08
E08000026	Coventry	2014_2015	2014	2015	387.3848	E08
E08000026	Coventry	2015_2016	2015	2016	465.5505	E08
E09000008	Croydon	2011_2012	2011	2012	87.52382	E09
E09000008	Croydon	2012_2013	2012	2013	215.5408	E09
E09000008	Croydon	2013_2014	2013	2014	368.3991	E09
E09000008	Croydon	2014_2015	2014	2015	470.2936	E09
E09000008	Croydon	2015_2016	2015	2016	562.171	E09
E10000006	Cumbria	2011_2012	2011	2012	63.69909	E10
E10000006	Cumbria	2012_2013	2012	2013	155.3138	E10
E10000006	Cumbria	2013_2014	2013	2014	261.779	E10
E10000006	Cumbria	2014_2015	2014	2015	340.1539	E10
E10000006	Cumbria	2015_2016	2015	2016	410.8556	E10
E06000005	Darlington	2011_2012	2011	2012	88.57331	E06
E06000005	Darlington	2012_2013	2012	2013	213.1159	E06
E06000005	Darlington	2013_2014	2013	2014	353.0609	E06
E06000005	Darlington	2014_2015	2014	2015	453.3683	E06
E06000005	Darlington	2015_2016	2015	2016	545.212	E06
W0600000	Denbighshi	2011_2012	2011	2012	90.65678	W06
W0600000	Denbighshi	2012_2013	2012	2013	216.4654	W06
W0600000	Denbighshi	2013_2014	2013	2014	344.9919	W06
W0600000	Denbighshi	2014_2015	2014	2015	453.0562	W06
W0600000	Denbighshi	2015_2016	2015	2016	550.2805	W06
E06000015	Derby UA	2011_2012	2011	2012	81.10015	E06
E06000015	Derby UA	2012_2013	2012	2013	195.3146	E06
E06000015	Derby UA	2013_2014	2013	2014	333.2738	E06
E06000015	Derby UA	2014_2015	2014	2015	427.546	E06
E06000015	Derby UA	2015_2016	2015	2016	512.4772	E06
E10000007	Derbyshire	2011_2012	2011	2012	65.75749	E10
E10000007	Derbyshire	2012_2013	2012	2013	160.6779	E10
E10000007	Derbyshire	2013_2014	2013	2014	270.707	E10
E10000007	Derbyshire	2014_2015	2014	2015	347.1842	E10
E10000007	Derbyshire	2015_2016	2015	2016	415.691	E10
E10000008	Devon	2011_2012	2011	2012	68.49664	E10
E10000008	Devon	2012_2013	2012	2013	166.2527	E10
E10000008	Devon	2013_2014	2013	2014	274.5258	E10
E10000008	Devon	2014_2015	2014	2015	349.0348	E10
E10000008	Devon	2015_2016	2015	2016	414.9039	E10
E08000017	Doncaster	2011_2012	2011	2012	87.01232	E08
E08000017	Doncaster	2012_2013	2012	2013	208.5108	E08
E08000017	Doncaster	2013_2014	2013	2014	336.98	E08
E08000017	Doncaster	2014_2015	2014	2015	437.6844	E08

E08000017	Doncaster	2015_2016	2015	2016	528.5096	E08
E08000027	Dudley	2011_2012	2011	2012	74.38458	E08
E08000027	Dudley	2012_2013	2012	2013	178.79	E08
E08000027	Dudley	2013_2014	2013	2014	306.7509	E08
E08000027	Dudley	2014_2015	2014	2015	392.4521	E08
E08000027	Dudley	2015_2016	2015	2016	470.0937	E08
S12000006	Dumfries a	2011_2012	2011	2012	71.78942	S12
S12000006	Dumfries a	2012_2013	2012	2013	171.2004	S12
S12000006	Dumfries a	2013_2014	2013	2014	268.5162	S12
S12000006	Dumfries a	2014_2015	2014	2015	351.5755	S12
S12000006	Dumfries a	2015_2016	2015	2016	426.3305	S12
S12000042	Dundee Cit	2011_2012	2011	2012	78.92878	S12
S12000042	Dundee Cit	2012_2013	2012	2013	187.0052	S12
S12000042	Dundee Cit	2013_2014	2013	2014	295.7824	S12
S12000042	Dundee Cit	2014_2015	2014	2015	395.0974	S12
S12000042	Dundee Cit	2015_2016	2015	2016	483.9326	S12
E09000009	Ealing	2011_2012	2011	2012	88.67816	E09
E09000009	Ealing	2012_2013	2012	2013	218.869	E09
E09000009	Ealing	2013_2014	2013	2014	373.5146	E09
E09000009	Ealing	2014_2015	2014	2015	480.3226	E09
E09000009	Ealing	2015_2016	2015	2016	572.7478	E09
S12000008	East Ayrshi	2011_2012	2011	2012	78.29522	S12
S12000008	East Ayrshi	2012_2013	2012	2013	186.6181	S12
S12000008	East Ayrshi	2013_2014	2013	2014	297.8404	S12
S12000008	East Ayrshi	2014_2015	2014	2015	394.1829	S12
S12000008	East Ayrshi	2015_2016	2015	2016	480.2441	S12
S12000045	East Dunba	2011_2012	2011	2012	45.11555	S12
S12000045	East Dunba	2012_2013	2012	2013	113.4637	S12
S12000045	East Dunba	2013_2014	2013	2014	193.9345	S12
S12000045	East Dunba	2014_2015	2014	2015	247.472	S12
S12000045	East Dunba	2015_2016	2015	2016	293.8032	S12
S12000010	East Lothia	2011_2012	2011	2012	60.23269	S12
S12000010	East Lothia	2012_2013	2012	2013	147.031	S12
S12000010	East Lothia	2013_2014	2013	2014	239.1261	S12
S12000010	East Lothia	2014_2015	2014	2015	308.9715	S12
S12000010	East Lothia	2015_2016	2015	2016	370.3586	S12
S12000011	East Renfre	2011_2012	2011	2012	47.15831	S12
S12000011	East Renfre	2012_2013	2012	2013	119.3695	S12
S12000011	East Renfre	2013_2014	2013	2014	204.8206	S12
S12000011	East Renfre	2014_2015	2014	2015	260.1475	S12
S12000011	East Renfre	2015_2016	2015	2016	307.9012	S12
E06000011	East Riding	2011_2012	2011	2012	65.71637	E06
E06000011	East Riding	2012_2013	2012	2013	160.8697	E06
E06000011	East Riding	2013_2014	2013	2014	271.136	E06
E06000011	East Riding	2014_2015	2014	2015	344.1262	E06
E06000011	East Riding	2015_2016	2015	2016	410.442	E06
E10000011	East Sussex	2011_2012	2011	2012	77.29533	E10

E10000011	East Sussex	2012_2013	2012	2013	188.7922	E10
E10000011	East Sussex	2013_2014	2013	2014	300.3655	E10
E10000011	East Sussex	2014_2015	2014	2015	386.6827	E10
E10000011	East Sussex	2015_2016	2015	2016	463.7754	E10
S12000036	Edinburgh,	2011_2012	2011	2012	53.65873	S12
S12000036	Edinburgh,	2012_2013	2012	2013	131.0394	S12
S12000036	Edinburgh,	2013_2014	2013	2014	210.4447	S12
S12000036	Edinburgh,	2014_2015	2014	2015	275.4693	S12
S12000036	Edinburgh,	2015_2016	2015	2016	332.5815	S12
S12000013	Eilean Siar	2011_2012	2011	2012	53.13102	S12
S12000013	Eilean Siar	2012_2013	2012	2013	129.1117	S12
S12000013	Eilean Siar	2013_2014	2013	2014	212.6279	S12
S12000013	Eilean Siar	2014_2015	2014	2015	276.2572	S12
S12000013	Eilean Siar	2015_2016	2015	2016	332.366	S12
E09000010	Enfield	2011_2012	2011	2012	105.7879	E09
E09000010	Enfield	2012_2013	2012	2013	257.4645	E09
E09000010	Enfield	2013_2014	2013	2014	434.726	E09
E09000010	Enfield	2014_2015	2014	2015	563.9041	E09
E09000010	Enfield	2015_2016	2015	2016	678.1522	E09
E10000012	Essex	2011_2012	2011	2012	60.91859	E10
E10000012	Essex	2012_2013	2012	2013	151.1829	E10
E10000012	Essex	2013_2014	2013	2014	265.5663	E10
E10000012	Essex	2014_2015	2014	2015	333.3042	E10
E10000012	Essex	2015_2016	2015	2016	393.9132	E10
S12000014	Falkirk	2011_2012	2011	2012	60.81667	S12
S12000014	Falkirk	2012_2013	2012	2013	147.0408	S12
S12000014	Falkirk	2013_2014	2013	2014	241.7619	S12
S12000014	Falkirk	2014_2015	2014	2015	316.6654	S12
S12000014	Falkirk	2015_2016	2015	2016	382.8048	S12
S12000015	Fife	2011_2012	2011	2012	68.53825	S12
S12000015	Fife	2012_2013	2012	2013	164.4218	S12
S12000015	Fife	2013_2014	2013	2014	265.5455	S12
S12000015	Fife	2014_2015	2014	2015	349.0818	S12
S12000015	Fife	2015_2016	2015	2016	423.0476	S12
W0600000	Flintshire /	2011_2012	2011	2012	68.85043	W06
W0600000	Flintshire /	2012_2013	2012	2013	166.9392	W06
W0600000	Flintshire /	2013_2014	2013	2014	276.2567	W06
W0600000	Flintshire /	2014_2015	2014	2015	355.4558	W06
W0600000	Flintshire /	2015_2016	2015	2016	425.7557	W06
E08000037	Gateshead	2011_2012	2011	2012	76.09877	E08
E08000037	Gateshead	2012_2013	2012	2013	181.8797	E08
E08000037	Gateshead	2013_2014	2013	2014	311.96	E08
E08000037	Gateshead	2014_2015	2014	2015	403.8914	E08
E08000037	Gateshead	2015_2016	2015	2016	486.9812	E08
S12000046	Glasgow Ci	2011_2012	2011	2012	77.35911	S12
S12000046	Glasgow Ci	2012_2013	2012	2013	182.0734	S12
S12000046	Glasgow Ci	2013_2014	2013	2014	291.5607	S12

S12000046	Glasgow Ci	2014_2015	2014	2015	392.763	S12
S12000046	Glasgow Ci	2015_2016	2015	2016	483.002	S12
E10000013	Gloucester	2011_2012	2011	2012	58.00083	E10
E10000013	Gloucester	2012_2013	2012	2013	142.8868	E10
E10000013	Gloucester	2013_2014	2013	2014	236.1382	E10
E10000013	Gloucester	2014_2015	2014	2015	300.6168	E10
E10000013	Gloucester	2015_2016	2015	2016	356.744	E10
E09000011	Greenwich	2011_2012	2011	2012	69.86399	E09
E09000011	Greenwich	2012_2013	2012	2013	170.5425	E09
E09000011	Greenwich	2013_2014	2013	2014	306.0336	E09
E09000011	Greenwich	2014_2015	2014	2015	389.7764	E09
E09000011	Greenwich	2015_2016	2015	2016	464.437	E09
W0600000	Gwynedd /	2011_2012	2011	2012	67.3773	W06
W0600000	Gwynedd /	2012_2013	2012	2013	161.4785	W06
W0600000	Gwynedd /	2013_2014	2013	2014	265.643	W06
W0600000	Gwynedd /	2014_2015	2014	2015	343.209	W06
W0600000	Gwynedd /	2015_2016	2015	2016	412.5636	W06
E09000012	Hackney	2011_2012	2011	2012	96.89539	E09
E09000012	Hackney	2012_2013	2012	2013	236.6337	E09
E09000012	Hackney	2013_2014	2013	2014	403.5617	E09
E09000012	Hackney	2014_2015	2014	2015	513.198	E09
E09000012	Hackney	2015_2016	2015	2016	611.7458	E09
E06000006	Halton UA	2011_2012	2011	2012	87.78203	E06
E06000006	Halton UA	2012_2013	2012	2013	210.0593	E06
E06000006	Halton UA	2013_2014	2013	2014	373.4558	E06
E06000006	Halton UA	2014_2015	2014	2015	479.059	E06
E06000006	Halton UA	2015_2016	2015	2016	575.225	E06
E09000013	Hammersm	2011_2012	2011	2012	62.18839	E09
E09000013	Hammersm	2012_2013	2012	2013	155.0806	E09
E09000013	Hammersm	2013_2014	2013	2014	270.4147	E09
E09000013	Hammersm	2014_2015	2014	2015	350.0511	E09
E09000013	Hammersm	2015_2016	2015	2016	416.4965	E09
E10000014	Hampshire	2011_2012	2011	2012	49.9169	E10
E10000014	Hampshire	2012_2013	2012	2013	125.7972	E10
E10000014	Hampshire	2013_2014	2013	2014	218.7928	E10
E10000014	Hampshire	2014_2015	2014	2015	274.5981	E10
E10000014	Hampshire	2015_2016	2015	2016	323.444	E10
E09000014	Haringey	2011_2012	2011	2012	94.8552	E09
E09000014	Haringey	2012_2013	2012	2013	231.4105	E09
E09000014	Haringey	2013_2014	2013	2014	404.9458	E09
E09000014	Haringey	2014_2015	2014	2015	520.7446	E09
E09000014	Haringey	2015_2016	2015	2016	624.0131	E09
E09000015	Harrow	2011_2012	2011	2012	73.07471	E09
E09000015	Harrow	2012_2013	2012	2013	181.5304	E09
E09000015	Harrow	2013_2014	2013	2014	315.8757	E09
E09000015	Harrow	2014_2015	2014	2015	402.8296	E09
E09000015	Harrow	2015_2016	2015	2016	480.0909	E09

E06000001	Hartlepool	2011_2012	2011	2012	104.0031	E06
E06000001	Hartlepool	2012_2013	2012	2013	249.5384	E06
E06000001	Hartlepool	2013_2014	2013	2014	423.2226	E06
E06000001	Hartlepool	2014_2015	2014	2015	547.9994	E06
E06000001	Hartlepool	2015_2016	2015	2016	661.5406	E06
E09000016	Havering	2011_2012	2011	2012	62.21169	E09
E09000016	Havering	2012_2013	2012	2013	154.9131	E09
E09000016	Havering	2013_2014	2013	2014	270.2131	E09
E09000016	Havering	2014_2015	2014	2015	341.6109	E09
E09000016	Havering	2015_2016	2015	2016	404.4364	E09
E06000019	Herefordsh	2011_2012	2011	2012	61.65162	E06
E06000019	Herefordsh	2012_2013	2012	2013	148.7496	E06
E06000019	Herefordsh	2013_2014	2013	2014	249.4239	E06
E06000019	Herefordsh	2014_2015	2014	2015	317.273	E06
E06000019	Herefordsh	2015_2016	2015	2016	378.605	E06
E10000015	Hertfordsh	2011_2012	2011	2012	52.74683	E10
E10000015	Hertfordsh	2012_2013	2012	2013	133.5155	E10
E10000015	Hertfordsh	2013_2014	2013	2014	238.4713	E10
E10000015	Hertfordsh	2014_2015	2014	2015	295.3566	E10
E10000015	Hertfordsh	2015_2016	2015	2016	344.164	E10
S12000017	Highland	2011_2012	2011	2012	56.98902	S12
S12000017	Highland	2012_2013	2012	2013	137.5004	S12
S12000017	Highland	2013_2014	2013	2014	222.6453	S12
S12000017	Highland	2014_2015	2014	2015	290.3541	S12
S12000017	Highland	2015_2016	2015	2016	350.3888	S12
E09000017	Hillingdon	2011_2012	2011	2012	68.14352	E09
E09000017	Hillingdon	2012_2013	2012	2013	169.735	E09
E09000017	Hillingdon	2013_2014	2013	2014	295.1577	E09
E09000017	Hillingdon	2014_2015	2014	2015	374.4186	E09
E09000017	Hillingdon	2015_2016	2015	2016	444.623	E09
E09000018	Hounslow	2011_2012	2011	2012	73.98907	E09
E09000018	Hounslow	2012_2013	2012	2013	182.6401	E09
E09000018	Hounslow	2013_2014	2013	2014	305.3449	E09
E09000018	Hounslow	2014_2015	2014	2015	389.7819	E09
E09000018	Hounslow	2015_2016	2015	2016	464.2712	E09
S12000018	Inverclyde	2011_2012	2011	2012	84.00818	S12
S12000018	Inverclyde	2012_2013	2012	2013	199.3092	S12
S12000018	Inverclyde	2013_2014	2013	2014	317.9642	S12
S12000018	Inverclyde	2014_2015	2014	2015	425.0313	S12
S12000018	Inverclyde	2015_2016	2015	2016	521.2841	S12
W0600000	Isle of Angl	2011_2012	2011	2012	79.98089	W06
W0600000	Isle of Angl	2012_2013	2012	2013	191.8444	W06
W0600000	Isle of Angl	2013_2014	2013	2014	313.0173	W06
W0600000	Isle of Angl	2014_2015	2014	2015	406.5701	W06
W0600000	Isle of Angl	2015_2016	2015	2016	490.1358	W06
E06000046	Isle of Wigl	2011_2012	2011	2012	89.853	E06
E06000046	Isle of Wigl	2012_2013	2012	2013	215.8595	E06

E06000046	Isle of Wigh	2013_2014	2013	2014	355.6224	E06
E06000046	Isle of Wigh	2014_2015	2014	2015	457.8334	E06
E06000046	Isle of Wigh	2015_2016	2015	2016	552.557	E06
E09000019	Islington	2011_2012	2011	2012	65.96751	E09
E09000019	Islington	2012_2013	2012	2013	162.5472	E09
E09000019	Islington	2013_2014	2013	2014	284.0343	E09
E09000019	Islington	2014_2015	2014	2015	362.3668	E09
E09000019	Islington	2015_2016	2015	2016	431.2927	E09
E09000020	Kensington	2011_2012	2011	2012	100.8035	E09
E09000020	Kensington	2012_2013	2012	2013	254.5367	E09
E09000020	Kensington	2013_2014	2013	2014	378.5497	E09
E09000020	Kensington	2014_2015	2014	2015	474.3583	E09
E09000020	Kensington	2015_2016	2015	2016	558.046	E09
E10000016	Kent	2011_2012	2011	2012	68.47966	E10
E10000016	Kent	2012_2013	2012	2013	168.0549	E10
E10000016	Kent	2013_2014	2013	2014	283.8984	E10
E10000016	Kent	2014_2015	2014	2015	359.0171	E10
E10000016	Kent	2015_2016	2015	2016	425.66	E10
E06000010	Kingston up	2011_2012	2011	2012	101.5889	E06
E06000010	Kingston up	2012_2013	2012	2013	240.498	E06
E06000010	Kingston up	2013_2014	2013	2014	406.8375	E06
E06000010	Kingston up	2014_2015	2014	2015	526.2089	E06
E06000010	Kingston up	2015_2016	2015	2016	636.1959	E06
E09000021	Kingston up	2011_2012	2011	2012	50.90879	E09
E09000021	Kingston up	2012_2013	2012	2013	130.4368	E09
E09000021	Kingston up	2013_2014	2013	2014	221.1397	E09
E09000021	Kingston up	2014_2015	2014	2015	278.7797	E09
E09000021	Kingston up	2015_2016	2015	2016	328.2755	E09
E08000034	Kirklees	2011_2012	2011	2012	81.43883	E08
E08000034	Kirklees	2012_2013	2012	2013	196.3203	E08
E08000034	Kirklees	2013_2014	2013	2014	325.5896	E08
E08000034	Kirklees	2014_2015	2014	2015	415.8729	E08
E08000034	Kirklees	2015_2016	2015	2016	498.095	E08
E08000011	Knowsley	2011_2012	2011	2012	104.7576	E08
E08000011	Knowsley	2012_2013	2012	2013	249.6947	E08
E08000011	Knowsley	2013_2014	2013	2014	448.2368	E08
E08000011	Knowsley	2014_2015	2014	2015	577.0588	E08
E08000011	Knowsley	2015_2016	2015	2016	694.519	E08
E09000022	Lambeth	2011_2012	2011	2012	69.84183	E09
E09000022	Lambeth	2012_2013	2012	2013	171.6019	E09
E09000022	Lambeth	2013_2014	2013	2014	307.8343	E09
E09000022	Lambeth	2014_2015	2014	2015	392.5687	E09
E09000022	Lambeth	2015_2016	2015	2016	467.9786	E09
E10000017	Lancashire	2011_2012	2011	2012	75.7339	E10
E10000017	Lancashire	2012_2013	2012	2013	183.6775	E10
E10000017	Lancashire	2013_2014	2013	2014	307.3335	E10
E10000017	Lancashire	2014_2015	2014	2015	396.5726	E10

E10000017	Lancashire	2015_2016	2015	2016	476.0441	E10
E08000035	Leeds	2011_2012	2011	2012	69.98047	E08
E08000035	Leeds	2012_2013	2012	2013	169.1837	E08
E08000035	Leeds	2013_2014	2013	2014	294.7991	E08
E08000035	Leeds	2014_2015	2014	2015	375.9268	E08
E08000035	Leeds	2015_2016	2015	2016	449.6377	E08
E06000016	Leicester U	2011_2012	2011	2012	87.90466	E06
E06000016	Leicester U	2012_2013	2012	2013	208.5236	E06
E06000016	Leicester U	2013_2014	2013	2014	352.1728	E06
E06000016	Leicester U	2014_2015	2014	2015	452.2524	E06
E06000016	Leicester U	2015_2016	2015	2016	543.63	E06
E10000018	Leicestersh	2011_2012	2011	2012	52.8282	E10
E10000018	Leicestersh	2012_2013	2012	2013	130.3692	E10
E10000018	Leicestersh	2013_2014	2013	2014	221.8026	E10
E10000018	Leicestersh	2014_2015	2014	2015	279.1385	E10
E10000018	Leicestersh	2015_2016	2015	2016	328.4738	E10
E09000023	Lewisham	2011_2012	2011	2012	90.62292	E09
E09000023	Lewisham	2012_2013	2012	2013	222.6721	E09
E09000023	Lewisham	2013_2014	2013	2014	375.4043	E09
E09000023	Lewisham	2014_2015	2014	2015	478.0396	E09
E09000023	Lewisham	2015_2016	2015	2016	570.058	E09
E10000019	Lincolnshir	2011_2012	2011	2012	73.11142	E10
E10000019	Lincolnshir	2012_2013	2012	2013	177.1806	E10
E10000019	Lincolnshir	2013_2014	2013	2014	293.9485	E10
E10000019	Lincolnshir	2014_2015	2014	2015	374.185	E10
E10000019	Lincolnshir	2015_2016	2015	2016	447.1539	E10
E08000012	Liverpool	2011_2012	2011	2012	90.915	E08
E08000012	Liverpool	2012_2013	2012	2013	216.3451	E08
E08000012	Liverpool	2013_2014	2013	2014	369.2255	E08
E08000012	Liverpool	2014_2015	2014	2015	481.1081	E08
E08000012	Liverpool	2015_2016	2015	2016	583.3594	E08
E06000032	Luton UA	2011_2012	2011	2012	88.02977	E06
E06000032	Luton UA	2012_2013	2012	2013	212.3224	E06
E06000032	Luton UA	2013_2014	2013	2014	336.0346	E06
E06000032	Luton UA	2014_2015	2014	2015	434.0418	E06
E06000032	Luton UA	2015_2016	2015	2016	521.4864	E06
E08000003	Mancheste	2011_2012	2011	2012	84.15078	E08
E08000003	Mancheste	2012_2013	2012	2013	199.8684	E08
E08000003	Mancheste	2013_2014	2013	2014	342.4639	E08
E08000003	Mancheste	2014_2015	2014	2015	441.2113	E08
E08000003	Mancheste	2015_2016	2015	2016	530.878	E08
E06000035	Medway U.	2011_2012	2011	2012	76.83063	E06
E06000035	Medway U.	2012_2013	2012	2013	187.5455	E06
E06000035	Medway U.	2013_2014	2013	2014	322.4677	E06
E06000035	Medway U.	2014_2015	2014	2015	410.9195	E06
E06000035	Medway U.	2015_2016	2015	2016	491.5243	E06
W0600002	Merthyr Ty	2011_2012	2011	2012	90.22496	W06

W0600002	Merthyr Ty	2012_2013	2012	2013	213.8996	W06
W0600002	Merthyr Ty	2013_2014	2013	2014	362.9749	W06
W0600002	Merthyr Ty	2014_2015	2014	2015	478.7023	W06
W0600002	Merthyr Ty	2015_2016	2015	2016	582.2181	W06
E09000024	Merton	2011_2012	2011	2012	65.27303	E09
E09000024	Merton	2012_2013	2012	2013	163.2869	E09
E09000024	Merton	2013_2014	2013	2014	273.2258	E09
E09000024	Merton	2014_2015	2014	2015	350.4371	E09
E09000024	Merton	2015_2016	2015	2016	417.8523	E09
E06000002	Middlesbro	2011_2012	2011	2012	108.8495	E06
E06000002	Middlesbro	2012_2013	2012	2013	258.9609	E06
E06000002	Middlesbro	2013_2014	2013	2014	446.4475	E06
E06000002	Middlesbro	2014_2015	2014	2015	574.0277	E06
E06000002	Middlesbro	2015_2016	2015	2016	691.7521	E06
S12000019	Midlothian	2011_2012	2011	2012	63.72545	S12
S12000019	Midlothian	2012_2013	2012	2013	153.701	S12
S12000019	Midlothian	2013_2014	2013	2014	248.3889	S12
S12000019	Midlothian	2014_2015	2014	2015	323.9582	S12
S12000019	Midlothian	2015_2016	2015	2016	390.6091	S12
E06000042	Milton Key	2011_2012	2011	2012	73.7827	E06
E06000042	Milton Key	2012_2013	2012	2013	182.0209	E06
E06000042	Milton Key	2013_2014	2013	2014	310.2412	E06
E06000042	Milton Key	2014_2015	2014	2015	391.4944	E06
E06000042	Milton Key	2015_2016	2015	2016	464.6192	E06
W0600002	Monmouth	2011_2012	2011	2012	54.99152	W06
W0600002	Monmouth	2012_2013	2012	2013	134.822	W06
W0600002	Monmouth	2013_2014	2013	2014	229.4532	W06
W0600002	Monmouth	2014_2015	2014	2015	294.9695	W06
W0600002	Monmouth	2015_2016	2015	2016	352.7346	W06
S12000020	Moray	2011_2012	2011	2012	49.77375	S12
S12000020	Moray	2012_2013	2012	2013	120.5102	S12
S12000020	Moray	2013_2014	2013	2014	195.15	S12
S12000020	Moray	2014_2015	2014	2015	253.2761	S12
S12000020	Moray	2015_2016	2015	2016	304.9009	S12
W0600001	Neath Port	2011_2012	2011	2012	85.90359	W06
W0600001	Neath Port	2012_2013	2012	2013	204.3636	W06
W0600001	Neath Port	2013_2014	2013	2014	342.8182	W06
W0600001	Neath Port	2014_2015	2014	2015	450.4337	W06
W0600001	Neath Port	2015_2016	2015	2016	546.6068	W06
E08000021	Newcastle	2011_2012	2011	2012	67.66913	E08
E08000021	Newcastle	2012_2013	2012	2013	160.8605	E08
E08000021	Newcastle	2013_2014	2013	2014	292.6444	E08
E08000021	Newcastle	2014_2015	2014	2015	373.9846	E08
E08000021	Newcastle	2015_2016	2015	2016	448.7411	E08
E09000025	Newham	2011_2012	2011	2012	86.04421	E09
E09000025	Newham	2012_2013	2012	2013	206.6794	E09
E09000025	Newham	2013_2014	2013	2014	346.8109	E09

E09000025	Newham	2014_2015	2014	2015	449.7902	E09
E09000025	Newham	2015_2016	2015	2016	541.8637	E09
W0600002	Newport /	2011_2012	2011	2012	82.50648	W06
W0600002	Newport /	2012_2013	2012	2013	197.758	W06
W0600002	Newport /	2013_2014	2013	2014	329.8979	W06
W0600002	Newport /	2014_2015	2014	2015	428.1403	W06
W0600002	Newport /	2015_2016	2015	2016	515.9339	W06
E10000020	Norfolk	2011_2012	2011	2012	67.54369	E10
E10000020	Norfolk	2012_2013	2012	2013	163.6096	E10
E10000020	Norfolk	2013_2014	2013	2014	272.3758	E10
E10000020	Norfolk	2014_2015	2014	2015	348.9586	E10
E10000020	Norfolk	2015_2016	2015	2016	416.6541	E10
S12000021	North Ayrsl	2011_2012	2011	2012	86.07615	S12
S12000021	North Ayrsl	2012_2013	2012	2013	205.1617	S12
S12000021	North Ayrsl	2013_2014	2013	2014	327.0629	S12
S12000021	North Ayrsl	2014_2015	2014	2015	434.4278	S12
S12000021	North Ayrsl	2015_2016	2015	2016	530.7258	S12
E06000012	North East	2011_2012	2011	2012	97.4502	E06
E06000012	North East	2012_2013	2012	2013	233.2271	E06
E06000012	North East	2013_2014	2013	2014	395.6131	E06
E06000012	North East	2014_2015	2014	2015	509.5522	E06
E06000012	North East	2015_2016	2015	2016	615.1063	E06
S12000044	North Lana	2011_2012	2011	2012	73.4315	S12
S12000044	North Lana	2012_2013	2012	2013	175.4836	S12
S12000044	North Lana	2013_2014	2013	2014	284.6863	S12
S12000044	North Lana	2014_2015	2014	2015	377.3417	S12
S12000044	North Lana	2015_2016	2015	2016	459.8259	S12
E06000013	North Linco	2011_2012	2011	2012	70.28958	E06
E06000013	North Linco	2012_2013	2012	2013	170.4951	E06
E06000013	North Linco	2013_2014	2013	2014	289.0155	E06
E06000013	North Linco	2014_2015	2014	2015	370.9816	E06
E06000013	North Linco	2015_2016	2015	2016	444.7349	E06
E06000024	North Som	2011_2012	2011	2012	69.43402	E06
E06000024	North Som	2012_2013	2012	2013	170.7359	E06
E06000024	North Som	2013_2014	2013	2014	288.5537	E06
E06000024	North Som	2014_2015	2014	2015	369.4702	E06
E06000024	North Som	2015_2016	2015	2016	442.0231	E06
E08000022	North Tyne	2011_2012	2011	2012	76.21262	E08
E08000022	North Tyne	2012_2013	2012	2013	183.4302	E08
E08000022	North Tyne	2013_2014	2013	2014	309.7144	E08
E08000022	North Tyne	2014_2015	2014	2015	397.9357	E08
E08000022	North Tyne	2015_2016	2015	2016	477.5165	E08
E10000023	North York	2011_2012	2011	2012	57.90978	E10
E10000023	North York	2012_2013	2012	2013	143.0225	E10
E10000023	North York	2013_2014	2013	2014	238.2456	E10
E10000023	North York	2014_2015	2014	2015	305.4236	E10
E10000023	North York	2015_2016	2015	2016	365.5552	E10

E10000021	Northampt	2011_2012	2011	2012	67.49261	E10
E10000021	Northampt	2012_2013	2012	2013	165.4318	E10
E10000021	Northampt	2013_2014	2013	2014	282.7248	E10
E10000021	Northampt	2014_2015	2014	2015	356.4002	E10
E10000021	Northampt	2015_2016	2015	2016	420.3836	E10
E06000057	Northumbe	2011_2012	2011	2012	70.46279	E06
E06000057	Northumbe	2012_2013	2012	2013	169.9227	E06
E06000057	Northumbe	2013_2014	2013	2014	279.5025	E06
E06000057	Northumbe	2014_2015	2014	2015	361.5442	E06
E06000057	Northumbe	2015_2016	2015	2016	434.9615	E06
E06000018	Nottinghan	2011_2012	2011	2012	82.36135	E06
E06000018	Nottinghan	2012_2013	2012	2013	195.9559	E06
E06000018	Nottinghan	2013_2014	2013	2014	336.2689	E06
E06000018	Nottinghan	2014_2015	2014	2015	432.7387	E06
E06000018	Nottinghan	2015_2016	2015	2016	521.1075	E06
E10000024	Nottinghan	2011_2012	2011	2012	67.19457	E10
E10000024	Nottinghan	2012_2013	2012	2013	163.7925	E10
E10000024	Nottinghan	2013_2014	2013	2014	272.9209	E10
E10000024	Nottinghan	2014_2015	2014	2015	350.5247	E10
E10000024	Nottinghan	2015_2016	2015	2016	419.1243	E10
E08000004	Oldham	2011_2012	2011	2012	94.85766	E08
E08000004	Oldham	2012_2013	2012	2013	226.5231	E08
E08000004	Oldham	2013_2014	2013	2014	383.1313	E08
E08000004	Oldham	2014_2015	2014	2015	491.1921	E08
E08000004	Oldham	2015_2016	2015	2016	589.6502	E08
S12000023	Orkney Isla	2011_2012	2011	2012	50.33418	S12
S12000023	Orkney Isla	2012_2013	2012	2013	123.0189	S12
S12000023	Orkney Isla	2013_2014	2013	2014	198.7799	S12
S12000023	Orkney Isla	2014_2015	2014	2015	255.8207	S12
S12000023	Orkney Isla	2015_2016	2015	2016	306.4074	S12
E10000025	Oxfordshire	2011_2012	2011	2012	47.28683	E10
E10000025	Oxfordshire	2012_2013	2012	2013	119.385	E10
E10000025	Oxfordshire	2013_2014	2013	2014	204.7239	E10
E10000025	Oxfordshire	2014_2015	2014	2015	256.7745	E10
E10000025	Oxfordshire	2015_2016	2015	2016	302.1047	E10
W0600000	Pembrokes	2011_2012	2011	2012	86.61292	W06
W0600000	Pembrokes	2012_2013	2012	2013	207.6177	W06
W0600000	Pembrokes	2013_2014	2013	2014	328.3001	W06
W0600000	Pembrokes	2014_2015	2014	2015	423.9386	W06
W0600000	Pembrokes	2015_2016	2015	2016	509.9377	W06
S12000024	Perth and K	2011_2012	2011	2012	58.05455	S12
S12000024	Perth and K	2012_2013	2012	2013	140.7659	S12
S12000024	Perth and K	2013_2014	2013	2014	225.3819	S12
S12000024	Perth and K	2014_2015	2014	2015	291.3863	S12
S12000024	Perth and K	2015_2016	2015	2016	349.8966	S12
E06000031	Peterborou	2011_2012	2011	2012	92.52303	E06
E06000031	Peterborou	2012_2013	2012	2013	222.2562	E06

E06000031	Peterborou	2013_2014	2013	2014	378.0014	E06
E06000031	Peterborou	2014_2015	2014	2015	480.9114	E06
E06000031	Peterborou	2015_2016	2015	2016	574.0068	E06
E06000026	Plymouth U	2011_2012	2011	2012	78.57528	E06
E06000026	Plymouth U	2012_2013	2012	2013	188.8782	E06
E06000026	Plymouth U	2013_2014	2013	2014	319.9227	E06
E06000026	Plymouth U	2014_2015	2014	2015	412.1821	E06
E06000026	Plymouth U	2015_2016	2015	2016	496.6559	E06
E06000044	Portsmouth	2011_2012	2011	2012	71.29451	E06
E06000044	Portsmouth	2012_2013	2012	2013	172.2806	E06
E06000044	Portsmouth	2013_2014	2013	2014	289.3565	E06
E06000044	Portsmouth	2014_2015	2014	2015	371.3571	E06
E06000044	Portsmouth	2015_2016	2015	2016	446.0795	E06
W0600002	Powys / Po	2011_2012	2011	2012	68.78914	W06
W0600002	Powys / Po	2012_2013	2012	2013	165.3033	W06
W0600002	Powys / Po	2013_2014	2013	2014	270.3542	W06
W0600002	Powys / Po	2014_2015	2014	2015	348.2431	W06
W0600002	Powys / Po	2015_2016	2015	2016	417.85	W06
E06000038	Reading UA	2011_2012	2011	2012	68.33492	E06
E06000038	Reading UA	2012_2013	2012	2013	168.0884	E06
E06000038	Reading UA	2013_2014	2013	2014	286.8046	E06
E06000038	Reading UA	2014_2015	2014	2015	366.0263	E06
E06000038	Reading UA	2015_2016	2015	2016	437.3178	E06
E09000026	Redbridge	2011_2012	2011	2012	74.14295	E09
E09000026	Redbridge	2012_2013	2012	2013	183.902	E09
E09000026	Redbridge	2013_2014	2013	2014	305.0702	E09
E09000026	Redbridge	2014_2015	2014	2015	393.4406	E09
E09000026	Redbridge	2015_2016	2015	2016	470.1962	E09
E06000003	Redcar and	2011_2012	2011	2012	91.05101	E06
E06000003	Redcar and	2012_2013	2012	2013	217.6244	E06
E06000003	Redcar and	2013_2014	2013	2014	378.0912	E06
E06000003	Redcar and	2014_2015	2014	2015	487.4657	E06
E06000003	Redcar and	2015_2016	2015	2016	587.8992	E06
S12000038	Renfrewshi	2011_2012	2011	2012	70.87523	S12
S12000038	Renfrewshi	2012_2013	2012	2013	170.3195	S12
S12000038	Renfrewshi	2013_2014	2013	2014	274.7393	S12
S12000038	Renfrewshi	2014_2015	2014	2015	361.7495	S12
S12000038	Renfrewshi	2015_2016	2015	2016	439.2441	S12
W0600001	Rhondda, C	2011_2012	2011	2012	86.89907	W06
W0600001	Rhondda, C	2012_2013	2012	2013	206.8119	W06
W0600001	Rhondda, C	2013_2014	2013	2014	343.3426	W06
W0600001	Rhondda, C	2014_2015	2014	2015	453.5819	W06
W0600001	Rhondda, C	2015_2016	2015	2016	552.4114	W06
E09000027	Richmond u	2011_2012	2011	2012	42.83081	E09
E09000027	Richmond u	2012_2013	2012	2013	114.6798	E09
E09000027	Richmond u	2013_2014	2013	2014	208.3509	E09
E09000027	Richmond u	2014_2015	2014	2015	257.5561	E09

E09000027	Richmond (	2015_2016	2015	2016	298.8123	E09
E08000005	Rochdale	2011_2012	2011	2012	97.56661	E08
E08000005	Rochdale	2012_2013	2012	2013	233.8267	E08
E08000005	Rochdale	2013_2014	2013	2014	402.5135	E08
E08000005	Rochdale	2014_2015	2014	2015	515.9183	E08
E08000005	Rochdale	2015_2016	2015	2016	619.6276	E08
E08000018	Rotherham	2011_2012	2011	2012	81.33625	E08
E08000018	Rotherham	2012_2013	2012	2013	194.5591	E08
E08000018	Rotherham	2013_2014	2013	2014	329.9527	E08
E08000018	Rotherham	2014_2015	2014	2015	425.3231	E08
E08000018	Rotherham	2015_2016	2015	2016	511.4363	E08
E06000017	Rutland UA	2011_2012	2011	2012	39.73806	E06
E06000017	Rutland UA	2012_2013	2012	2013	100.1583	E06
E06000017	Rutland UA	2013_2014	2013	2014	181.0951	E06
E06000017	Rutland UA	2014_2015	2014	2015	222.5884	E06
E06000017	Rutland UA	2015_2016	2015	2016	259.2031	E06
E08000006	Salford	2011_2012	2011	2012	88.17277	E08
E08000006	Salford	2012_2013	2012	2013	210.9117	E08
E08000006	Salford	2013_2014	2013	2014	359.0845	E08
E08000006	Salford	2014_2015	2014	2015	460.6216	E08
E08000006	Salford	2015_2016	2015	2016	553.0848	E08
E08000028	Sandwell	2011_2012	2011	2012	94.01326	E08
E08000028	Sandwell	2012_2013	2012	2013	223.8123	E08
E08000028	Sandwell	2013_2014	2013	2014	367.375	E08
E08000028	Sandwell	2014_2015	2014	2015	476.4003	E08
E08000028	Sandwell	2015_2016	2015	2016	573.9159	E08
S12000026	Scottish Bo	2011_2012	2011	2012	61.55528	S12
S12000026	Scottish Bo	2012_2013	2012	2013	148.6558	S12
S12000026	Scottish Bo	2013_2014	2013	2014	237.7867	S12
S12000026	Scottish Bo	2014_2015	2014	2015	308.7638	S12
S12000026	Scottish Bo	2015_2016	2015	2016	371.7214	S12
E08000014	Sefton	2011_2012	2011	2012	85.13622	E08
E08000014	Sefton	2012_2013	2012	2013	204.5042	E08
E08000014	Sefton	2013_2014	2013	2014	352.6822	E08
E08000014	Sefton	2014_2015	2014	2015	454.2812	E08
E08000014	Sefton	2015_2016	2015	2016	547.2967	E08
E08000019	Sheffield	2011_2012	2011	2012	64.99349	E08
E08000019	Sheffield	2012_2013	2012	2013	155.6163	E08
E08000019	Sheffield	2013_2014	2013	2014	273.2432	E08
E08000019	Sheffield	2014_2015	2014	2015	349.8444	E08
E08000019	Sheffield	2015_2016	2015	2016	419.5984	E08
S12000027	Shetland Is	2011_2012	2011	2012	34.56737	S12
S12000027	Shetland Is	2012_2013	2012	2013	87.7504	S12
S12000027	Shetland Is	2013_2014	2013	2014	154.8392	S12
S12000027	Shetland Is	2014_2015	2014	2015	197.4697	S12
S12000027	Shetland Is	2015_2016	2015	2016	234.2236	S12
E06000051	Shropshire	2011_2012	2011	2012	56.38804	E06

E06000051	Shropshire	2012_2013	2012	2013	137.1428	E06
E06000051	Shropshire	2013_2014	2013	2014	227.3664	E06
E06000051	Shropshire	2014_2015	2014	2015	290.9778	E06
E06000051	Shropshire	2015_2016	2015	2016	347.396	E06
E06000039	Slough UA	2011_2012	2011	2012	90.33242	E06
E06000039	Slough UA	2012_2013	2012	2013	221.7839	E06
E06000039	Slough UA	2013_2014	2013	2014	372.2273	E06
E06000039	Slough UA	2014_2015	2014	2015	471.1845	E06
E06000039	Slough UA	2015_2016	2015	2016	559.0294	E06
E08000029	Solihull	2011_2012	2011	2012	61.3823	E08
E08000029	Solihull	2012_2013	2012	2013	152.1842	E08
E08000029	Solihull	2013_2014	2013	2014	256.7534	E08
E08000029	Solihull	2014_2015	2014	2015	327.4622	E08
E08000029	Solihull	2015_2016	2015	2016	389.7537	E08
E10000027	Somerset	2011_2012	2011	2012	66.77979	E10
E10000027	Somerset	2012_2013	2012	2013	162.7979	E10
E10000027	Somerset	2013_2014	2013	2014	274.067	E10
E10000027	Somerset	2014_2015	2014	2015	348.8533	E10
E10000027	Somerset	2015_2016	2015	2016	415.485	E10
S12000028	South Ayrsl	2011_2012	2011	2012	70.91894	S12
S12000028	South Ayrsl	2012_2013	2012	2013	170.6086	S12
S12000028	South Ayrsl	2013_2014	2013	2014	274.8336	S12
S12000028	South Ayrsl	2014_2015	2014	2015	362.8444	S12
S12000028	South Ayrsl	2015_2016	2015	2016	441.4147	S12
E06000025	South Glou	2011_2012	2011	2012	52.70222	E06
E06000025	South Glou	2012_2013	2012	2013	130.8712	E06
E06000025	South Glou	2013_2014	2013	2014	224.8901	E06
E06000025	South Glou	2014_2015	2014	2015	282.6407	E06
E06000025	South Glou	2015_2016	2015	2016	334.1729	E06
S12000029	South Lana	2011_2012	2011	2012	66.87773	S12
S12000029	South Lana	2012_2013	2012	2013	161.4579	S12
S12000029	South Lana	2013_2014	2013	2014	264.3536	S12
S12000029	South Lana	2014_2015	2014	2015	347.6759	S12
S12000029	South Lana	2015_2016	2015	2016	421.4482	S12
E08000023	South Tyne	2011_2012	2011	2012	85.73185	E08
E08000023	South Tyne	2012_2013	2012	2013	203.7199	E08
E08000023	South Tyne	2013_2014	2013	2014	375.2772	E08
E08000023	South Tyne	2014_2015	2014	2015	480.0661	E08
E08000023	South Tyne	2015_2016	2015	2016	576.9819	E08
E06000045	Southampt	2011_2012	2011	2012	67.45828	E06
E06000045	Southampt	2012_2013	2012	2013	162.818	E06
E06000045	Southampt	2013_2014	2013	2014	281.9783	E06
E06000045	Southampt	2014_2015	2014	2015	359.6175	E06
E06000045	Southampt	2015_2016	2015	2016	430.7182	E06
E06000033	Southend-c	2011_2012	2011	2012	88.30113	E06
E06000033	Southend-c	2012_2013	2012	2013	214.9983	E06
E06000033	Southend-c	2013_2014	2013	2014	361.7324	E06

E06000033	Southend-c	2014_2015	2014	2015	466.0958	E06
E06000033	Southend-c	2015_2016	2015	2016	561.745	E06
E09000028	Southwark	2011_2012	2011	2012	65.16012	E09
E09000028	Southwark	2012_2013	2012	2013	159.4239	E09
E09000028	Southwark	2013_2014	2013	2014	290.7219	E09
E09000028	Southwark	2014_2015	2014	2015	369.367	E09
E09000028	Southwark	2015_2016	2015	2016	439.4935	E09
E08000013	St. Helens	2011_2012	2011	2012	85.15798	E08
E08000013	St. Helens	2012_2013	2012	2013	204.5872	E08
E08000013	St. Helens	2013_2014	2013	2014	363.0553	E08
E08000013	St. Helens	2014_2015	2014	2015	465.4973	E08
E08000013	St. Helens	2015_2016	2015	2016	558.5602	E08
E10000028	Staffordshi	2011_2012	2011	2012	58.59376	E10
E10000028	Staffordshi	2012_2013	2012	2013	143.9814	E10
E10000028	Staffordshi	2013_2014	2013	2014	247.3548	E10
E10000028	Staffordshi	2014_2015	2014	2015	315.3467	E10
E10000028	Staffordshi	2015_2016	2015	2016	376.7111	E10
S12000030	Stirling	2011_2012	2011	2012	47.36954	S12
S12000030	Stirling	2012_2013	2012	2013	115.6196	S12
S12000030	Stirling	2013_2014	2013	2014	193.3899	S12
S12000030	Stirling	2014_2015	2014	2015	252.7094	S12
S12000030	Stirling	2015_2016	2015	2016	304.7416	S12
E08000007	Stockport	2011_2012	2011	2012	68.41689	E08
E08000007	Stockport	2012_2013	2012	2013	167.1313	E08
E08000007	Stockport	2013_2014	2013	2014	275.1101	E08
E08000007	Stockport	2014_2015	2014	2015	353.5679	E08
E08000007	Stockport	2015_2016	2015	2016	423.3023	E08
E06000004	Stockton-o	2011_2012	2011	2012	82.11397	E06
E06000004	Stockton-o	2012_2013	2012	2013	198.0409	E06
E06000004	Stockton-o	2013_2014	2013	2014	341.5422	E06
E06000004	Stockton-o	2014_2015	2014	2015	437.5855	E06
E06000004	Stockton-o	2015_2016	2015	2016	525.2553	E06
E06000021	Stoke-on-T	2011_2012	2011	2012	95.02264	E06
E06000021	Stoke-on-T	2012_2013	2012	2013	226.044	E06
E06000021	Stoke-on-T	2013_2014	2013	2014	392.4898	E06
E06000021	Stoke-on-T	2014_2015	2014	2015	503.8728	E06
E06000021	Stoke-on-T	2015_2016	2015	2016	606.0086	E06
E10000029	Suffolk	2011_2012	2011	2012	62.02006	E10
E10000029	Suffolk	2012_2013	2012	2013	151.9664	E10
E10000029	Suffolk	2013_2014	2013	2014	254.7527	E10
E10000029	Suffolk	2014_2015	2014	2015	324.4919	E10
E10000029	Suffolk	2015_2016	2015	2016	386.8645	E10
E08000024	Sunderlanc	2011_2012	2011	2012	89.45692	E08
E08000024	Sunderlanc	2012_2013	2012	2013	213.5771	E08
E08000024	Sunderlanc	2013_2014	2013	2014	360.534	E08
E08000024	Sunderlanc	2014_2015	2014	2015	468.6795	E08
E08000024	Sunderlanc	2015_2016	2015	2016	566.3101	E08

E10000030	Surrey	2011_2012	2011	2012	43.10772	E10
E10000030	Surrey	2012_2013	2012	2013	112.1567	E10
E10000030	Surrey	2013_2014	2013	2014	201.4376	E10
E10000030	Surrey	2014_2015	2014	2015	249.398	E10
E10000030	Surrey	2015_2016	2015	2016	289.7704	E10
E09000029	Sutton	2011_2012	2011	2012	63.40611	E09
E09000029	Sutton	2012_2013	2012	2013	158.615	E09
E09000029	Sutton	2013_2014	2013	2014	275.5576	E09
E09000029	Sutton	2014_2015	2014	2015	347.3846	E09
E09000029	Sutton	2015_2016	2015	2016	411.1069	E09
W0600001	Swansea /	2011_2012	2011	2012	76.00648	W06
W0600001	Swansea /	2012_2013	2012	2013	181.5542	W06
W0600001	Swansea /	2013_2014	2013	2014	299.6757	W06
W0600001	Swansea /	2014_2015	2014	2015	390.8943	W06
W0600001	Swansea /	2015_2016	2015	2016	472.5128	W06
E06000030	Swindon U	2011_2012	2011	2012	67.30642	E06
E06000030	Swindon U	2012_2013	2012	2013	164.8366	E06
E06000030	Swindon U	2013_2014	2013	2014	282.2466	E06
E06000030	Swindon U	2014_2015	2014	2015	357.3558	E06
E06000030	Swindon U	2015_2016	2015	2016	424.9843	E06
E08000008	Tameside	2011_2012	2011	2012	91.00589	E08
E08000008	Tameside	2012_2013	2012	2013	218.2407	E08
E08000008	Tameside	2013_2014	2013	2014	372.6634	E08
E08000008	Tameside	2014_2015	2014	2015	478.5632	E08
E08000008	Tameside	2015_2016	2015	2016	575.0277	E08
E06000020	Telford anc	2011_2012	2011	2012	81.69673	E06
E06000020	Telford anc	2012_2013	2012	2013	196.4834	E06
E06000020	Telford anc	2013_2014	2013	2014	339.9127	E06
E06000020	Telford anc	2014_2015	2014	2015	433.9341	E06
E06000020	Telford anc	2015_2016	2015	2016	519.3934	E06
W0600001	The Vale of	2011_2012	2011	2012	72.23051	W06
W0600001	The Vale of	2012_2013	2012	2013	175.8625	W06
W0600001	The Vale of	2013_2014	2013	2014	287.2826	W06
W0600001	The Vale of	2014_2015	2014	2015	370.2661	W06
W0600001	The Vale of	2015_2016	2015	2016	444.6288	W06
E06000034	Thurrock U	2011_2012	2011	2012	74.97569	E06
E06000034	Thurrock U	2012_2013	2012	2013	183.7593	E06
E06000034	Thurrock U	2013_2014	2013	2014	315.6628	E06
E06000034	Thurrock U	2014_2015	2014	2015	399.0518	E06
E06000034	Thurrock U	2015_2016	2015	2016	474.2939	E06
E06000027	Torbay UA	2011_2012	2011	2012	109.4929	E06
E06000027	Torbay UA	2012_2013	2012	2013	261.7862	E06
E06000027	Torbay UA	2013_2014	2013	2014	427.7771	E06
E06000027	Torbay UA	2014_2015	2014	2015	555.9877	E06
E06000027	Torbay UA	2015_2016	2015	2016	675.5944	E06
W0600002	Torfaen / T	2011_2012	2011	2012	80.78854	W06
W0600002	Torfaen / T	2012_2013	2012	2013	193.4031	W06

W0600002	Torfaen / T	2013_2014	2013	2014	332.4333	W06
W0600002	Torfaen / T	2014_2015	2014	2015	431.0074	W06
W0600002	Torfaen / T	2015_2016	2015	2016	518.6772	W06
E09000030	Tower Ham	2011_2012	2011	2012	69.15692	E09
E09000030	Tower Ham	2012_2013	2012	2013	168.465	E09
E09000030	Tower Ham	2013_2014	2013	2014	285.2265	E09
E09000030	Tower Ham	2014_2015	2014	2015	369.7483	E09
E09000030	Tower Ham	2015_2016	2015	2016	440.7203	E09
E08000009	Trafford	2011_2012	2011	2012	60.35664	E08
E08000009	Trafford	2012_2013	2012	2013	148.9559	E08
E08000009	Trafford	2013_2014	2013	2014	254.6081	E08
E08000009	Trafford	2014_2015	2014	2015	324.4395	E08
E08000009	Trafford	2015_2016	2015	2016	385.9323	E08
E08000036	Wakefield	2011_2012	2011	2012	77.80253	E08
E08000036	Wakefield	2012_2013	2012	2013	186.0197	E08
E08000036	Wakefield	2013_2014	2013	2014	329.7957	E08
E08000036	Wakefield	2014_2015	2014	2015	421.1082	E08
E08000036	Wakefield	2015_2016	2015	2016	504.4633	E08
E08000030	Walsall	2011_2012	2011	2012	87.61058	E08
E08000030	Walsall	2012_2013	2012	2013	208.75	E08
E08000030	Walsall	2013_2014	2013	2014	367.039	E08
E08000030	Walsall	2014_2015	2014	2015	468.3432	E08
E08000030	Walsall	2015_2016	2015	2016	561.1913	E08
E09000031	Waltham F	2011_2012	2011	2012	85.84875	E09
E09000031	Waltham F	2012_2013	2012	2013	209.6572	E09
E09000031	Waltham F	2013_2014	2013	2014	355.2939	E09
E09000031	Waltham F	2014_2015	2014	2015	457.4347	E09
E09000031	Waltham F	2015_2016	2015	2016	548.3486	E09
E09000032	Wandswor	2011_2012	2011	2012	63.04026	E09
E09000032	Wandswor	2012_2013	2012	2013	160.057	E09
E09000032	Wandswor	2013_2014	2013	2014	266.673	E09
E09000032	Wandswor	2014_2015	2014	2015	337.6307	E09
E09000032	Wandswor	2015_2016	2015	2016	398.7198	E09
E06000007	Warringtor	2011_2012	2011	2012	62.11995	E06
E06000007	Warringtor	2012_2013	2012	2013	152.3176	E06
E06000007	Warringtor	2013_2014	2013	2014	257.0257	E06
E06000007	Warringtor	2014_2015	2014	2015	329.0365	E06
E06000007	Warringtor	2015_2016	2015	2016	392.8584	E06
E10000031	Warwicksh	2011_2012	2011	2012	57.87431	E10
E10000031	Warwicksh	2012_2013	2012	2013	143.6746	E10
E10000031	Warwicksh	2013_2014	2013	2014	248.6955	E10
E10000031	Warwicksh	2014_2015	2014	2015	314.6172	E10
E10000031	Warwicksh	2015_2016	2015	2016	372.8444	E10
E06000037	West Berks	2011_2012	2011	2012	45.9571	E06
E06000037	West Berks	2012_2013	2012	2013	117.3344	E06
E06000037	West Berks	2013_2014	2013	2014	210.3046	E06
E06000037	West Berks	2014_2015	2014	2015	261.5848	E06

E06000037	West Berks	2015_2016	2015	2016	306.0984	E06
S12000039	West Dunb	2011_2012	2011	2012	78.42495	S12
S12000039	West Dunb	2012_2013	2012	2013	185.7517	S12
S12000039	West Dunb	2013_2014	2013	2014	300.3372	S12
S12000039	West Dunb	2014_2015	2014	2015	401.603	S12
S12000039	West Dunb	2015_2016	2015	2016	492.0426	S12
S12000040	West Lothi	2011_2012	2011	2012	67.25993	S12
S12000040	West Lothi	2012_2013	2012	2013	162.7836	S12
S12000040	West Lothi	2013_2014	2013	2014	265.4445	S12
S12000040	West Lothi	2014_2015	2014	2015	346.7088	S12
S12000040	West Lothi	2015_2016	2015	2016	418.2766	S12
E10000032	West Susse	2011_2012	2011	2012	59.17834	E10
E10000032	West Susse	2012_2013	2012	2013	146.3287	E10
E10000032	West Susse	2013_2014	2013	2014	244.8314	E10
E10000032	West Susse	2014_2015	2014	2015	309.7747	E10
E10000032	West Susse	2015_2016	2015	2016	365.7669	E10
E09000033	Westminst	2011_2012	2011	2012	138.8023	E09
E09000033	Westminst	2012_2013	2012	2013	352.601	E09
E09000033	Westminst	2013_2014	2013	2014	512.4476	E09
E09000033	Westminst	2014_2015	2014	2015	642.0348	E09
E09000033	Westminst	2015_2016	2015	2016	753.3525	E09
E08000010	Wigan	2011_2012	2011	2012	76.87506	E08
E08000010	Wigan	2012_2013	2012	2013	184.5906	E08
E08000010	Wigan	2013_2014	2013	2014	323.1133	E08
E08000010	Wigan	2014_2015	2014	2015	415.1058	E08
E08000010	Wigan	2015_2016	2015	2016	498.6253	E08
E06000054	Wiltshire U	2011_2012	2011	2012	52.3725	E06
E06000054	Wiltshire U	2012_2013	2012	2013	129.1175	E06
E06000054	Wiltshire U	2013_2014	2013	2014	226.0256	E06
E06000054	Wiltshire U	2014_2015	2014	2015	283.4053	E06
E06000054	Wiltshire U	2015_2016	2015	2016	334.8727	E06
E06000040	Windsor ar	2011_2012	2011	2012	40.92195	E06
E06000040	Windsor ar	2012_2013	2012	2013	108.1541	E06
E06000040	Windsor ar	2013_2014	2013	2014	199.377	E06
E06000040	Windsor ar	2014_2015	2014	2015	246.1995	E06
E06000040	Windsor ar	2015_2016	2015	2016	285.6073	E06
E08000015	Wirral	2011_2012	2011	2012	92.16552	E08
E08000015	Wirral	2012_2013	2012	2013	222.0522	E08
E08000015	Wirral	2013_2014	2013	2014	383.3389	E08
E08000015	Wirral	2014_2015	2014	2015	493.4938	E08
E08000015	Wirral	2015_2016	2015	2016	594.2761	E08
E06000041	Wokinghan	2011_2012	2011	2012	35.84025	E06
E06000041	Wokinghan	2012_2013	2012	2013	95.7601	E06
E06000041	Wokinghan	2013_2014	2013	2014	174.456	E06
E06000041	Wokinghan	2014_2015	2014	2015	214.5759	E06
E06000041	Wokinghan	2015_2016	2015	2016	248.1859	E06
E08000031	Wolverhan	2011_2012	2011	2012	90.71217	E08

E08000031	Wolverhan	2012_2013	2012	2013	215.757	E08
E08000031	Wolverhan	2013_2014	2013	2014	385.2537	E08
E08000031	Wolverhan	2014_2015	2014	2015	493.2077	E08
E08000031	Wolverhan	2015_2016	2015	2016	593.0287	E08
E10000034	Worcesters	2011_2012	2011	2012	59.99445	E10
E10000034	Worcesters	2012_2013	2012	2013	146.9737	E10
E10000034	Worcesters	2013_2014	2013	2014	250.1024	E10
E10000034	Worcesters	2014_2015	2014	2015	318.367	E10
E10000034	Worcesters	2015_2016	2015	2016	379.8137	E10
W0600000	Wrexham /	2011_2012	2011	2012	77.95037	W06
W0600000	Wrexham /	2012_2013	2012	2013	187.5049	W06
W0600000	Wrexham /	2013_2014	2013	2014	310.7351	W06
W0600000	Wrexham /	2014_2015	2014	2015	399.8664	W06
W0600000	Wrexham /	2015_2016	2015	2016	479.0541	W06
E06000014	York UA	2011_2012	2011	2012	47.19522	E06
E06000014	York UA	2012_2013	2012	2013	116.0534	E06
E06000014	York UA	2013_2014	2013	2014	201.5015	E06
E06000014	York UA	2014_2015	2014	2015	253.5132	E06
E06000014	York UA	2015_2016	2015	2016	300.6348	E06
