# Supplementary material for: Social security cuts and life expectancy: a longitudinal analysis of local authorities in England, Scotland and Wales
Source: J Epidemiol Community Health. 2023 Nov 7;78(2):82–7. doi: 10.1136/jech-2023-220328 (PMC10850624; doi:10.1136/jech-2023-220328)
Supplement: Supplementary data [file jech-2023-220328supp002.pdf]

Supplementary Materials

S2: Fixed effects regression results – the effect of cumulative social security cuts on life expectancy at birth (2-year time lag)

sex	term	ex estimates	95% CI lower	95% CI upper	p-value
Female	(Intercept)	83.35	83.20	83.49	0.00
Female	Year (mortality)	0.09	0.04	0.14	0.00
Female	Social security loss	-0.08	-0.13	-0.03	0.00
Female	Scotland	-2.36	-2.49	-2.23	0.00
Female	Wales	-1.82	-2.15	-1.49	0.00
Female	Social security loss*Scotland	-0.06	-0.09	-0.02	0.00
Female	Social security loss*Wales	0.00	-0.03	0.03	0.96
Male	(Intercept)	80.15	79.97	80.33	0.00
Male	Year (mortality)	0.06	0.01	0.11	0.03
Male	Social security loss	-0.02	-0.08	0.04	0.52
Male	Scotland	-3.22	-3.43	-3.02	0.00
Male	Wales	-1.50	-1.74	-1.25	0.00
Male	Social security loss*Scotland	-0.08	-0.12	-0.05	0.00
Male	Social security loss*Wales	-0.06	-0.11	-0.02	0.01

S3: Fixed effects regression results - the effect of cumulative social security cuts on remaining life expectancy at age 65 years old.

sex	term	ex estimates	95% CI lower	95% CI upper	p-value
Female	(Intercept)	21.53	21.46	21 .600	0.00
Female	Year (mortality)	0.03	0.00	0.06	0.07
Female	Social security loss	-0.02	-0.05	0.01	0.18
Female	Scotland	-1.80	-1.91	-1.69	0.00
Female	Wales	-1.62	-1.96	-1.28	0.00
Female	Social security loss*Scotland	0.02	0.00	0.05	0.06
Female	Social security loss*Wales	-0.02	-0.05	0.00	0.05
Male	(Intercept)	18.77	18.51	19.03	0.00
Male	Year (mortality)	0.07	0.03	0.11	0.00
Male	Social security loss	-0.02	-0.06	0.02	0.40
Male	Scotland	-1.71	-2.02	-1.41	0.00
Male	Wales	-0.72	-1.03	-0.42	0.00
Male	Social security loss*Scotland	-0.02	-0.04	0.01	0.25
Male	Social security loss*Wales	-0.01	-0.04	0.02	0.66

S4: Lifespan variation at birth

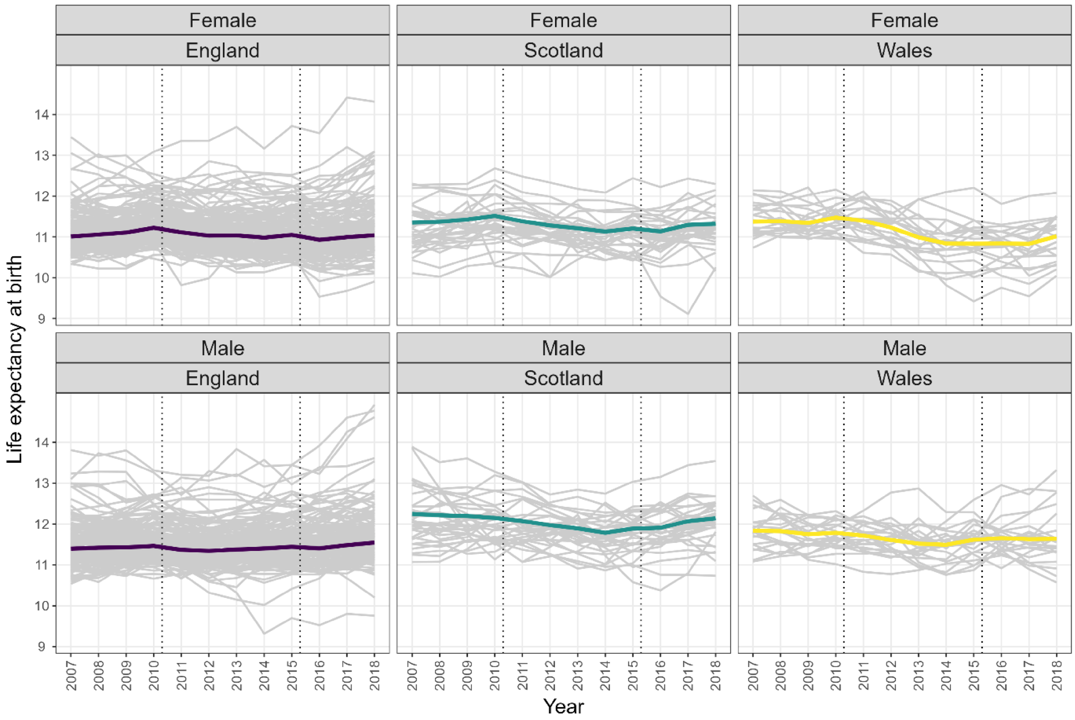

S5: Fixed effects regression results - the effect of cumulative social security cuts on lifespan variation at age 0.

sex	term	edagger estimates	95% CI lower	95% CI upper	p-value
Female	(Intercept)	11.17	11.00	11.34	0.00
Female	Year (mortality)	-0.04	-0.08	0.00	0.05
Female	Social security loss	0.03	-0.02	0.07	0.23
Female	Scotland	0.13	-0.05	0.30	0.15
Female	Wales	-0.24	-0.71	0.23	0.31
Female	Social security loss*Scotland	0.00	-0.04	0.03	0.73
Female	Social security loss*Wales	-0.08	-0.12	-0.04	0.00
Male	(Intercept)	11.04	10.83	11.24	0.00
Male	Year (mortality)	0.00	-0.04	0.04	0.87
Male	Social security loss	0.03	-0.01	0.06	0.19
Male	Scotland	0.77	0.55	1.00	0.00
Male	Wales	0.55	0.28	0.83	0.00
Male	Social security loss*Scotland	-0.05	-0.08	-0.02	0.00
Male	Social security loss*Wales	-0.01	-0.05	0.03	0.54
